# Estimation of alkali dosage and contact time for treating human excreta containing viruses as an emergency response: a systematic review

**DOI:** 10.3389/fpubh.2023.1286595

**Published:** 2023-11-10

**Authors:** Wakana Oishi, Daisuke Sano

**Affiliations:** ^1^Department of Civil and Environmental Engineering, Graduate School of Engineering, Tohoku University, Sendai, Japan; ^2^Department of Frontier Science for Advanced Environment, Graduate School of Environmental Studies, Tohoku University, Sendai, Japan

**Keywords:** sanitation, disinfection, slaked lime, alkaline treatment, viruses, machine learning

## Abstract

Water, sanitation, and hygiene provisions are essential during emergencies to prevent infectious disease outbreaks caused by improper human excreta management in settlements for people affected by natural disasters and conflicts. Human excreta disinfection is required when long-term containment in latrines is not feasible on-site. Alkali additives, including lime, are effective disinfectants for wastewater and faecal sludge containing large amounts of solid and dissolved organic matter. The aim of this study was to determine the minimum dose and contact time of alkali additives for treating virus-containing human excreta in emergency situations. We used literature data collected by searching Google Scholar and Web of Science. The date of the last search for each study was 31th May 2023. Only peer-reviewed articles that included disinfection practices in combination with quantitative data for the physicochemical data of a matrix and viral decay were selected for data extraction. Two reviewers independently collected data from each study. We extracted datasets from 14 studies that reported quantitative information about their disinfection tests, including viral decay over time, matrix types, and physicochemical properties. Three machine learning algorithms were applied to the collected dataset to determine the time required to achieve specified levels of virus inactivation under different environmental conditions. The best model was used to estimate the contact time to achieve a 3-log_10_ inactivation of RNA virus in wastewater and faeces. The most important variables for predicting the contact time were pH, temperature, and virus type. The estimated contact time for 3 log inactivation of RNA virus was <2 h at pH 12, which was achieved by adding 1.8 and 3.1% slaked lime to wastewater and faeces, respectively. The contact time decreased exponentially with the pH of the sludge and wastewater. In contrast, the pH of the sludge and wastewater increased linearly with the slaked lime dosage. Lime treatment is a promising measure where long-term containment in latrine is not feasible in densely populated areas, as 1 day is sufficient to inactivate viruses. The relationship we have identified between required contact time and lime dosage is useful for practitioners in determining appropriate treatment conditions of human waste.

## 1. Introduction

Safe water supply, sanitation, and hygiene (WASH) are essential in ensuring the health of populations affected by natural disasters (e.g., floods, earthquakes, and storms) and conflicts combined with adverse physical and social factors (e.g., poverty) (1). People affected by emergencies live in temporary shelters or camps, leave their homes, or reside near others. Outbreaks in settlements during emergencies have been caused by waterborne pathogens, including *Vibrio cholerae*, *Shigella*, hepatitis E virus, hepatitis A virus, norovirus, and rotavirus (2–7). Infectious disease outbreaks are associated with inadequate WASH facilities during natural disasters (8). Maintaining good toilet hygiene is necessary to prevent infectious gastroenteritis during temporary stays at evacuation shelters (9). Proper disposal of human excreta is a priority for reducing disease transmission through direct and indirect routes.

A common emergency measure is disposing in sewers; however, sewer system and treatment facilities are heavily damaged by earthquakes, stopping functioning for a long period (10). In the developing world, containing excreta in pit latrines is a common emergency measure to prevent environmental contamination. When the groundwater table is high, lack of space to dig pits, or rocky/sandy ground composition, constructing a pit latrine is not a suitable option (11). In such situations, excreta is retained in impermeable storage containers and left for extended periods to reduce the pathogen levels before moving the waste off-site for additional treatment and safe disposal. When it is not feasible to store waste owing to space constraints in densely populated spontaneous settlements, excreta containing infective pathogens are dumped in local streams or rivers and off-site without long-term storage, contaminating drinking water sources (12, 13).

The interest in on-site excreta disinfection is increasing (14). A chlorine solution (bleach) is commonly used to disinfect drinking water and clean contaminated environmental surfaces during emergencies. However, hypochlorite is ineffective in the presence of suspended solids and dissolved organic matter because of the formation of chloro-organic compounds and chloramines with low oxidation powers (15). A potential alternative is lime suspension. Lime preparations typically used for disinfection include slaked (Ca(OH)_2_) and burnt (CaO) lime. Lime has been used for treating high-organic-loading waste because it raises the sludge pH to >10, which is sufficient to inactivate viruses and bacteria (16). Lime is more efficacious than chlorine for inactivation of virus and bacteria (17). Chlorination is less effective for protozoa, including *Entamoeba histolytica* and *Giardia lamblia* (18). Although there are insufficient data to conclude on the effect of alkaline pH on the protozoan inactivation in sanitation-relevant matrices, alkaline treatment at pH > 9.25 increases the biocidal free ammonia (NH_3_) that can diffuse through the complex wall of protozoan oocysts (19).

The efficacy of liming in inactivating different groups of pathogens was investigated in a previous review. Gram-negative bacteria, including *Vibrio cholerae*, tend to be more sensitive to high pH than gram-positive bacteria, often requiring treatment times of less than 0.1 days to achieve a 2 log_10_ inactivation (16). To date, the effectiveness of disinfection in inactivating viruses has not been well characterised due to a lack of data on the inactivation of phage, which is used as an indicator of mammalian viruses, and consequently no concrete guidelines have yet been established for the alkaline treatment of virus-containing human waste. The extent of viral inactivation in human waste depends on contact time and pH. Contact time is shorter at higher pH, while more alkali additive is needed to maintain alkaline conditions. The inactivation efficacy can be described as a function of pH and quantity of alkali additives. However, disinfection efficacy is not determined by pH alone; it is affected by solid content, temperature, physical force (i.e., mixing), and the type of virus to be inactivated. In addition, the pH of the matrix is influenced by carbonate ions in the matrices and the buffer capacity. The treated matrices are readily neutralised due to calcium carbonate mineral formation. Various matrix properties should be considered when determining the appropriate additive dosage and contact time.

One promising approach is using an inactivation kinetics model that represents the log-reduction values (LRVs) of microorganisms as a function of variables related to the matrix and environmental conditions (20). Machine learning algorithms are advantageous for predicting LRVs because they can avoid overfitting training datasets using dimensional reduction, regularisation, and cross-validation (21). This study aimed to estimate the minimum contact time with alkali additives to achieve certain LRVs of viruses using datasets from the literature and three machine learning algorithms.

## 2. Methods

We conducted a systematic review following the PRISMA guidelines to compile quantitative data on virus inactivation in alkali-treated faecal matter (22). The following research question was used to guide our review: “*What is the contact time with alkali additives for treating a virus-containing matrix?*” Google Scholar and Web of Science were used for collecting relevant articles published between 1950 and May 2023. The keywords used were: (alkali) AND (virus) AND (inactivation OR disinfection) AND (biosolids OR sludge). The date of the last search for each study was 31th May 2023. Only peer-reviewed articles that included disinfection practices in combination with quantitative data for the physicochemical data of a matrix and viral decay were selected for data extraction. A single reviewer decided whether a study met the inclusion criteria for the review. The inclusion criteria for the papers were as follows: (1) published in English, (2) peer-reviewed, (3) not a review paper, (4) used alkali additives as disinfectants, and (5) containing the time-course change of virus infectivity and quantitative information on the physicochemical parameters of the matrices. Two reviewers independently extracted data from each study. Data extraction from one figure was performed using WebPlotDigitizer version 4.2 (23). Virus concentration or rate constant of a first-order kinetics based on infectivity assays was extracted. LRV of surrogate viruses was the outcome measure used in the synthesis of results.

A single reviewer calculated the times for *n* LRVs (T90 for *n* = 1, T99 for *n* = 2, T99.9 for *n* = 3, and T99.99 for *n* = 4), as described in a previous study (24). Briefly, the Hom model (25) was fitted to the time-series data of surrogate inactivation identified through a systematic review. It is used to describe the biphasic inactivation curves which include tailing (decay slowing with time) and shouldering (initial delay in decay), and used to determine survival rates after exposure. Hom’s model parameters, representing the first-order rate constant and degree of tailing-off, were estimated via the maximum likelihood method using the statistical software R (version 4.2.3). The R codes used in this study were provided in the supplementary information. The time required for *n* LRVs was then back-calculated using the fitted Hom model. We also collected quantitative and qualitative information on the matrix properties, virus types, and experimental settings, which were used as features in predicting time for *n* LRV.

Four individual models were developed to estimate T90, T99, T99.9, and T99.99. We employed three machine learning algorithms: random forest (RF), light GBM, and Automatic Relevance Determination (ARD) (scikit-learn version 1.2, Python). The model features were the physical and chemical properties of the matrix during the treatment, which are individual measurable properties or characteristics of a phenomenon. We split a dataset into train and test sets, and used resampling methods to handle the uncertainty in the representativeness of our dataset and estimated the performance of a modelling procedure on dataset not used in that procedure. Of the complete dataset, 80% was used to train the model and the remaining 20% to evaluate its prediction accuracy. The values of the hyperparameters were determined via a 5-fold cross validation. We conducted this set of data allocations and cross-validation 10 times. The mean square values (MSE) for predicting the test (MSEtest) and training (MSEtrain) data were calculated. We also estimated the ratio of MSEtest to MSEtrain, indicating the degree of overfitting to the training data. The best model was selected based on the mean value of MSE and the ratio of MSEtest to MSEtrain in the 10-time calculation.

We analysed the relationship between the alkali additive dose and pH of the matrix by applying regression analysis to the data collected in the systematic review. The dose of the alkali additives was back-calculated using a regression model. We used R version 4.2.3 (26) for statistical analyses and graphics creation. Finally, we estimated the appropriate contact times for certain LRVs and dose of alkali additives on sanitation-relevant materials based on the estimated dose-pH relationship in alkaline treatment and the best machine learning model among the three algorithms under the following assumption: RNA mammalian viruses; pH 10, 10.5, 11, 11.5, and 12; 10°C and 25°C; and wastewater and faeces. Although viruses are known to be present in high concentrations in wastewater and faeces, few studies have reported the concentration of infectious viral particles. The maximum concentrations of norovirus in faeces and wastewater are approximately 10^9^ copies/mL and 10^6^ copies/mL, respectively, by RT-qPCR (27–29), so we assumed a conservative infectious virus count in 1 mL of 10^6^ in the wastewater and 10^9^ in the faeces.

## 3. Results

**Figure 1:**
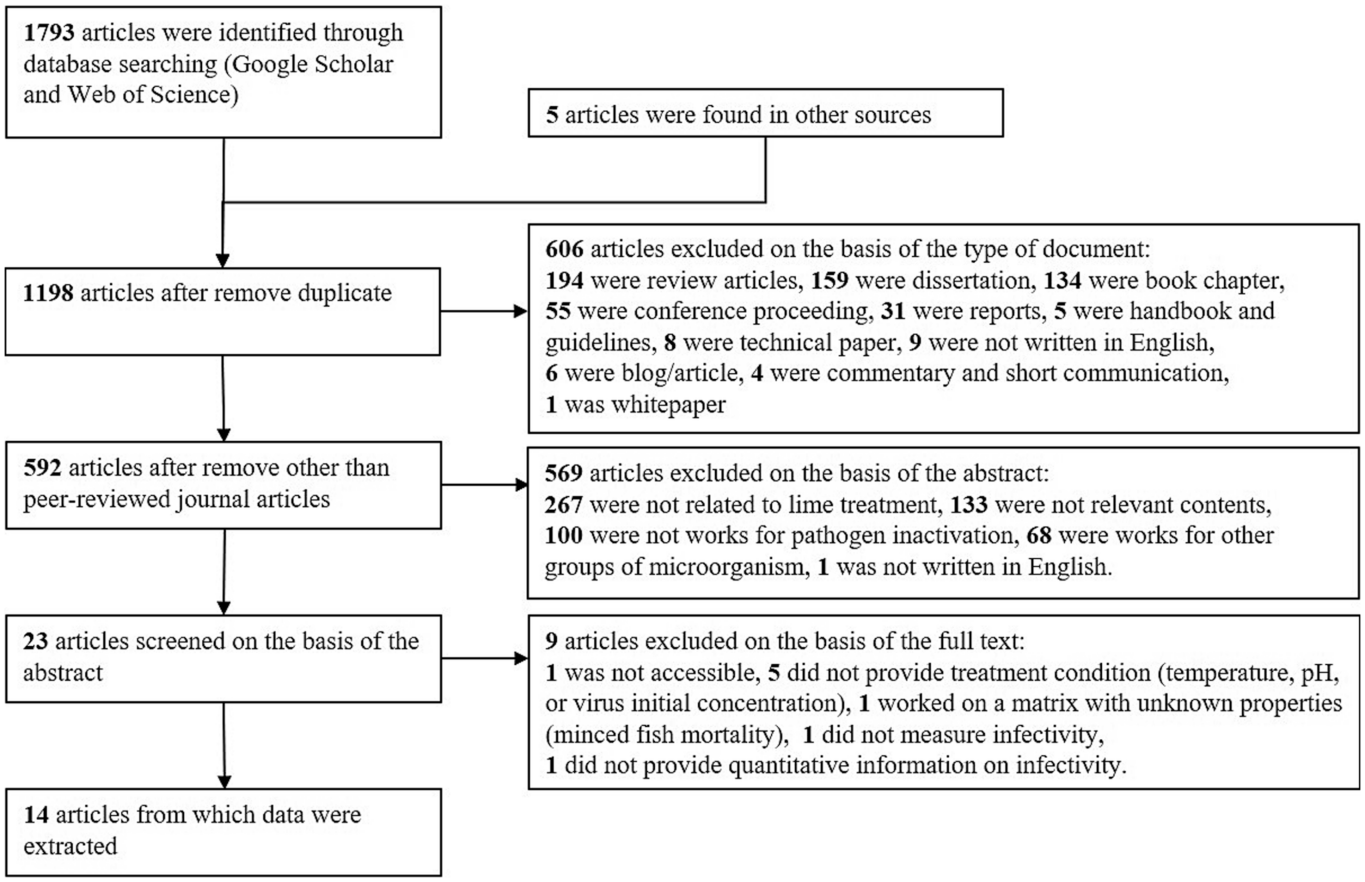
Flow diagram of the systematic review of time-course virus inactivation with alkali treatment.

The results of this systematic review are presented in Figure 1. We identified 1,793 records by searching Google Scholar and Web of Science. Five additional papers uncovered before this systematic review were included. Twenty-three studies were screened on the basis of the abstract, but 9 studies of these were not used for the further analysis for the following reason. Mignotte-Cadiergues et al. evaluated the effect of liming on the fate of somatic coliphages, F-RNA phages, *Bacteroides fragilis* phages, but was not accessible (30). Four studies did not report treatment conditions, including temperatures (31–33) and pH (34), or the initial viral concentration needed to calculate LRVs (32, 35). They were not applied to the dataset extraction to reduce the uncertainty due to the incomplete dataset in the further model development. One study used a minced fish mortality but was excluded to avoid introducing uncertainty arising from the unknown matrix property on virus persistence (36). Schmits et al. evaluated the efficacy of lime treatment of domestic duck slurry and showed that the M gene of A/H5N9 highly pathogenic avian influenza viruses was not detectable for 1 week at pH 12 (37). We did not extract their data because the viral genome quantity measured by M gene real-time reverse transcription PCR is not comparable to viral infectivity. Abu-Orf et al. reported that reovirus was completely inactivated in 12 days by 100 g burnt lime per kg biosolids (dry) and 50 g burnt lime per kg biosolids (dry) in combination with fly ash, but the virus concentration after disinfection was not reported (38).

Fourteen studies reported quantitative information on their disinfection tests, including the time-series decay of viruses, types of matrices, and physicochemical properties (Table 1). Synthesised matrices included water and buffer solutions, while non-synthesised matrices included wastewater, human faeces, human urine, faecal sludge, compost, activated sludge, and bovine serum. The alkali additives tested included slaked lime (17, 39–44), burnt lime (45), sodium hydroxide (36, 46–48), alkaline buffer (49), and ash (44, 50). The pH of the matrix after adding the alkali was 8.4–13.4. Only da Silva et al. monitored indigenous bacteriophages in faecal sludge, whereas the other studies propagated surrogates in a host, and inoculated in a matrix. Mammalian viruses used included adenovirus type 5 (39, 41), murine norovirus (43), avian influenza virus (44), Newcastle disease virus (44), and fish viruses (48). Mammalian viruses were used in five studies and bacteriophages in several others. Infectious virus was quantified as plaque-forming units (PFU) (for phages and rotavirus WA), most probable number (MPN) (49), or TCID_50_ infectious titer (41, 44). The infectious titer could variate among the three methods, but we assumed that inactivation rate is comparable, and thus the difference in quantification techniques was not considered in further analysis.

**Table 1:** Matrices, alkali additives, pH of the matrices after adding additives, and surrogate viruses in the included papers.

Reference	Matrix	Additive	pH	Surrogate	Number of experiments analysed further
Magri et al. (50)	Mixture of faeces, oyster shell, and urea (0–0.5%)	Ash	8.4–8.6	Coliphage MS2, ΦX174	6
Bean et al. (39)	Water	Ca(OH)_2_	12.0	Coliphage MS2, Adenovirus Type 5, Rotavirus WA	3
da Silva et al. (17)	Mixture of raw wastewater and faecal sludge	Ca(OH)_2_	11.9–13.4	Somatic coliphage, F + specific phages, *Bacteroides fragilis* phage	55
da Silva et al. (28)	Mixture of raw wastewater and faecal sludge	Ca(OH)_2_	12.8–12.9	Somatic coliphage	3
Hansen et al. (41)	Composted sludge, Raw sludge, Water	Ca(OH)_2_	12.0	Coliphage MS2, Adenovirus Type 5	8
Ogunyoku et al. (42)	Mixture of faeces and urine, Mixture of faeces and water	Ca(OH)_2_	12.3–12.7	Coliphage MS2	2
Oishi et al. (43)	Water	Ca(OH)_2_	12.2	Murine norovirus	1
Ruenphet et al. (44)	Fetal bovine serum, Water	Ca(OH)_2_, Charcoal ash	12.0–13.0	Avian influenza virus, Newcastle disease virus	23
Senecal et al. (51)	Mixture of faeces and ammonium carbonate buffer, buffer solutions, urine	Ca(OH)_2_	9.0–12.8	Coliphages T4, MS2, ΦX174	10
Hijikata et al. (45)	Compost	CaO	10.0–11.0	Coliphage MS2	3
Decrey et al. (49)	Phosphate buffer	–	10.0–12.0	Coliphages T4, MS2, ΦX174	9
Ruiz-Hernando et al. (46)	Activated sludge	NaOH	12.0	Somatic coliphage	1
Senecal et al. (47)	Glycine buffer	NaOH	10.5	Coliphage MS2, ΦX174	2
Dixon et al. (48)	Culture medium containing 3.75% bovine serum albumin	NaOH	12.0	Spring viremia of carp virus, Viral hemorrhagic septicemia virus, European sheatfish virus, European catfish virus, Infectious haematopoietic necrosis virus, Nervous necrosis virus	11

A total of 266 data points excluding time 0 were extracted (Supplementary Figure S1). Hom model was used to describe the biphasic inactivation curves which include tailing and shouldering as well as the monophasic inactivation curves. The mean deviation from the observed values was–0.0006, while the maximum deviation was 1.42 (Supplementary Figure S2). T90, T99, T99.9, and T99.99 were estimated using the Hom model fitted to the observed data. We did not extrapolate LRVs using a fitted decay curve for the experiment in which > *n* LRVs were not observed, to overestimating the LRVs achieved under the disinfection conditions. In the da Silva et al. experiments, > 2 LRVs were observed at the first sampling time, but no further LRVs were observed at the second sampling time (17). A steep increase in LRVs within the first sampling time was predicted by Hom’s models fitted to these data, which may lead to an overestimation of LRVs. When > *n* LRVs were observed at the first sampling time, we used the first sampling time as the time required for *n* LRVs to avoid underestimating the treatment time for virus inactivation. Five rate constants of a first-order kinetics were used to calculate T90, T99, T99.9, and T99.99 (51).In experiments in the seven studies, time series LRVs were not reported, but a post-disinfection virus concentration was available (17, 39, 41, 44, 46, 48, 49). They stopped sampling or could not follow the decay of the time series because a surrogate was inactivated too quickly down to the limit of detection. We included these two points of time-LRV data as follows: if > *n* LRVs were observed after disinfection, we used the exposure time as the time required for *n* LRVs. The size of the synthesised dataset used for further analysis was 136 for T90, 105 for T99, 77 for T99.9, and 57 for T99.99, respectively (Supplementary Table S1).

**Figure 2:**
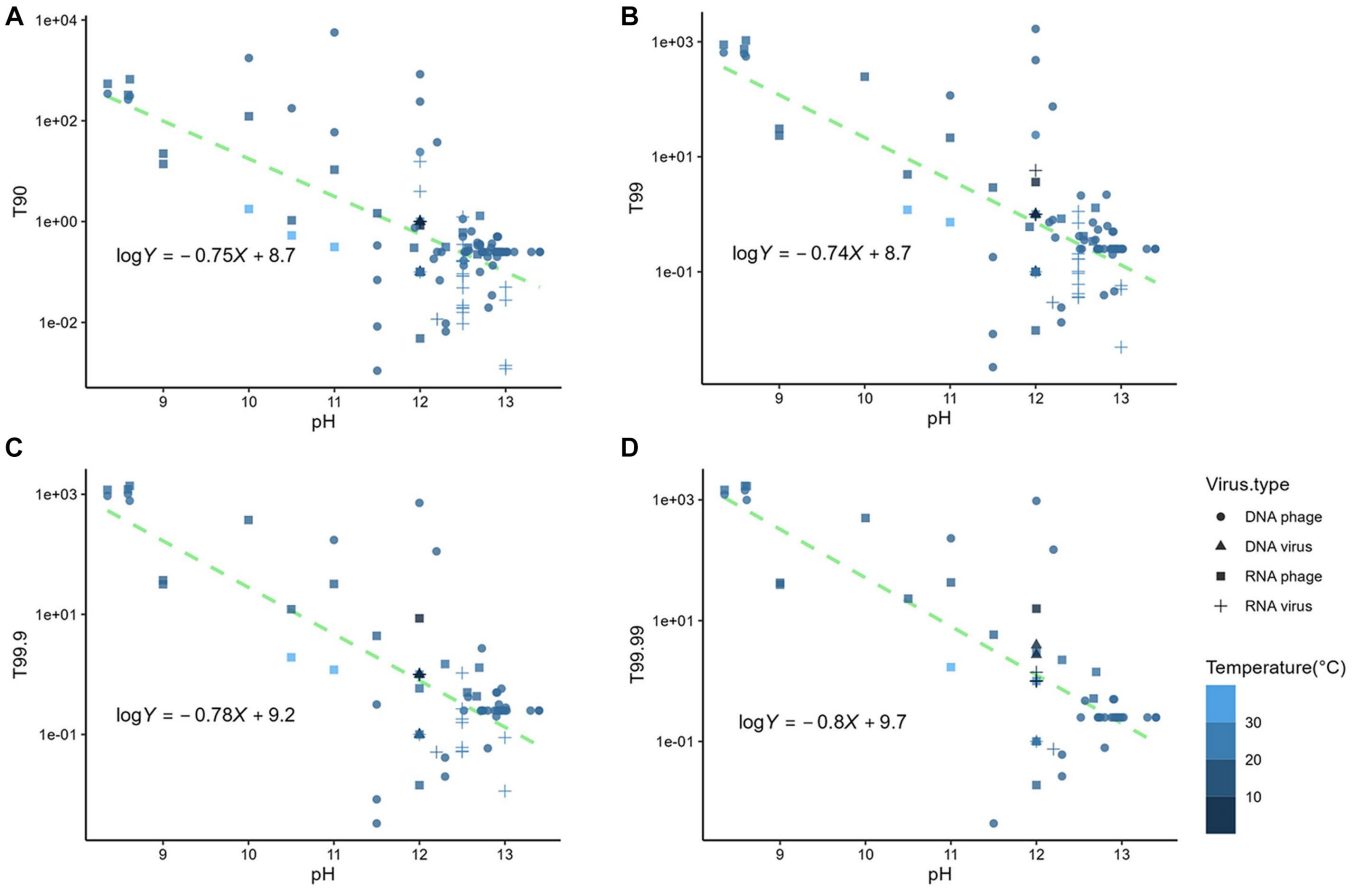
Relationship between the pH of the matrix and **(A)** T90, **(B)** T99, **(C)** T99.9, and **(D)** T99.99.

**Figure 3:**
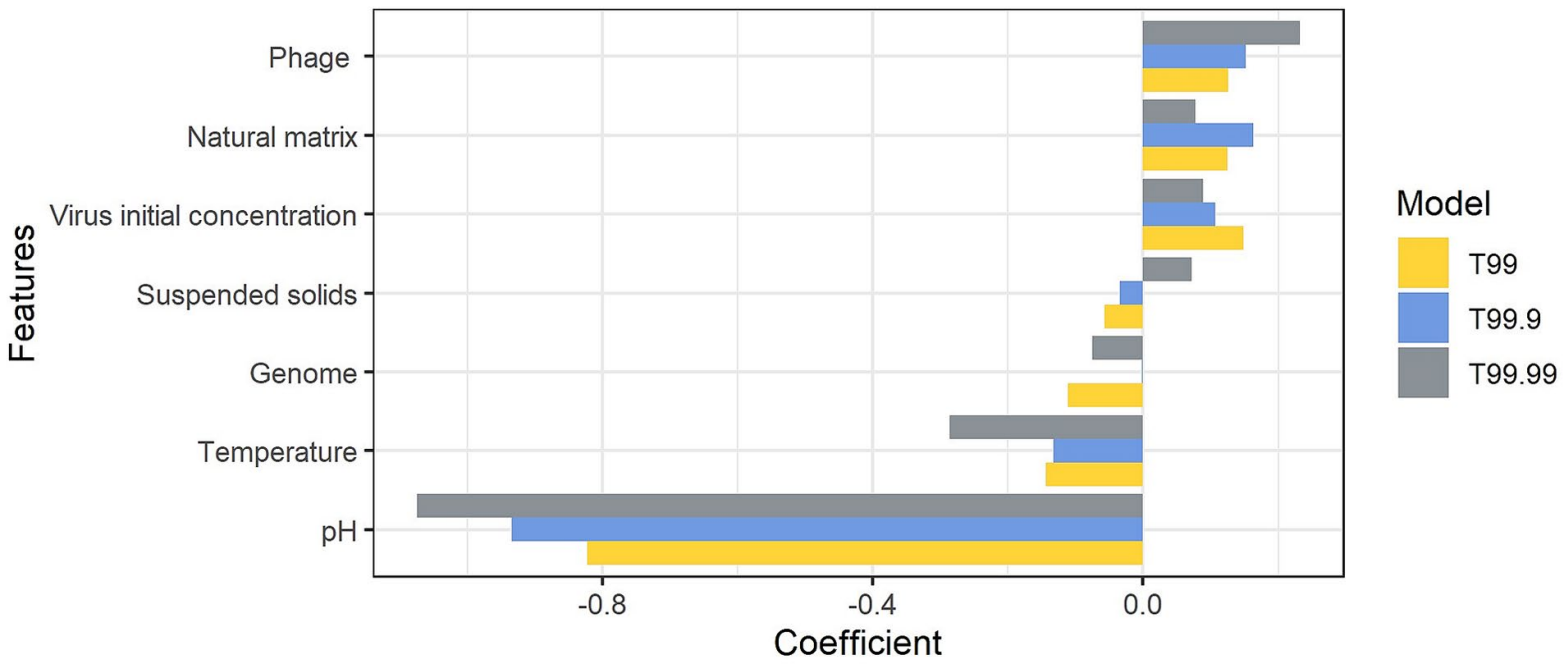
Coefficients of the Automatic Relevance Determination models for T99, T99.9, and T99.99.

The linear relationships were identified between the logarithmic times for inactivation and the pH of the matrix (*p* < 0.05) (Figure 2); however, the estimates based on the linear model deviated from the observed data. The mean absolute errors of T90, T99, T99.9, and T99.99 were 75 h, 69 h, 101 h, and 71 h, respectively. Those deviations indicated that times for *n* LRV could not be solely explained by pH. Other factors affected the disinfection efficiency. We employed other factors as input variables in the machine learning algorithms to minimize the deviations between the observed data and estimates. The variables used included the virus genome structure (RNA or DNA), virus type (phage or virus), matrix type (natural or synthesized), suspended solid contents (TSS; <12% as liquid, 12–30% as sludge, or > 30% as solid), pH, temperatures, and logarithmic initial virus concentration. MSEtest and MSEtrain by three machine learning models were shown in Supplementary Figure S3. The prediction accuracy of T90 was higher in the RF model, while those of T99, T99.9, and T99.99 were higher in the ARD model. The ratio of MSEtest to MSEtrain indicative for overfitting to training data was lowest in the ARD models, which indicated that the prediction by ARD models was robust to the unknown dataset. The pH value was the most important factor for contact time compared to the virus genome structure and abundance of suspended solids (Figure 3). The estimated contact time was shorter with a higher pH and temperature. Meanwhile, the coefficients were positive for virus type, matrix type, and initial virus concentration, indicating that the estimated contact time was longer for inactivating the phage in a natural matrix with a higher initial concentration.

**Figure 4:**
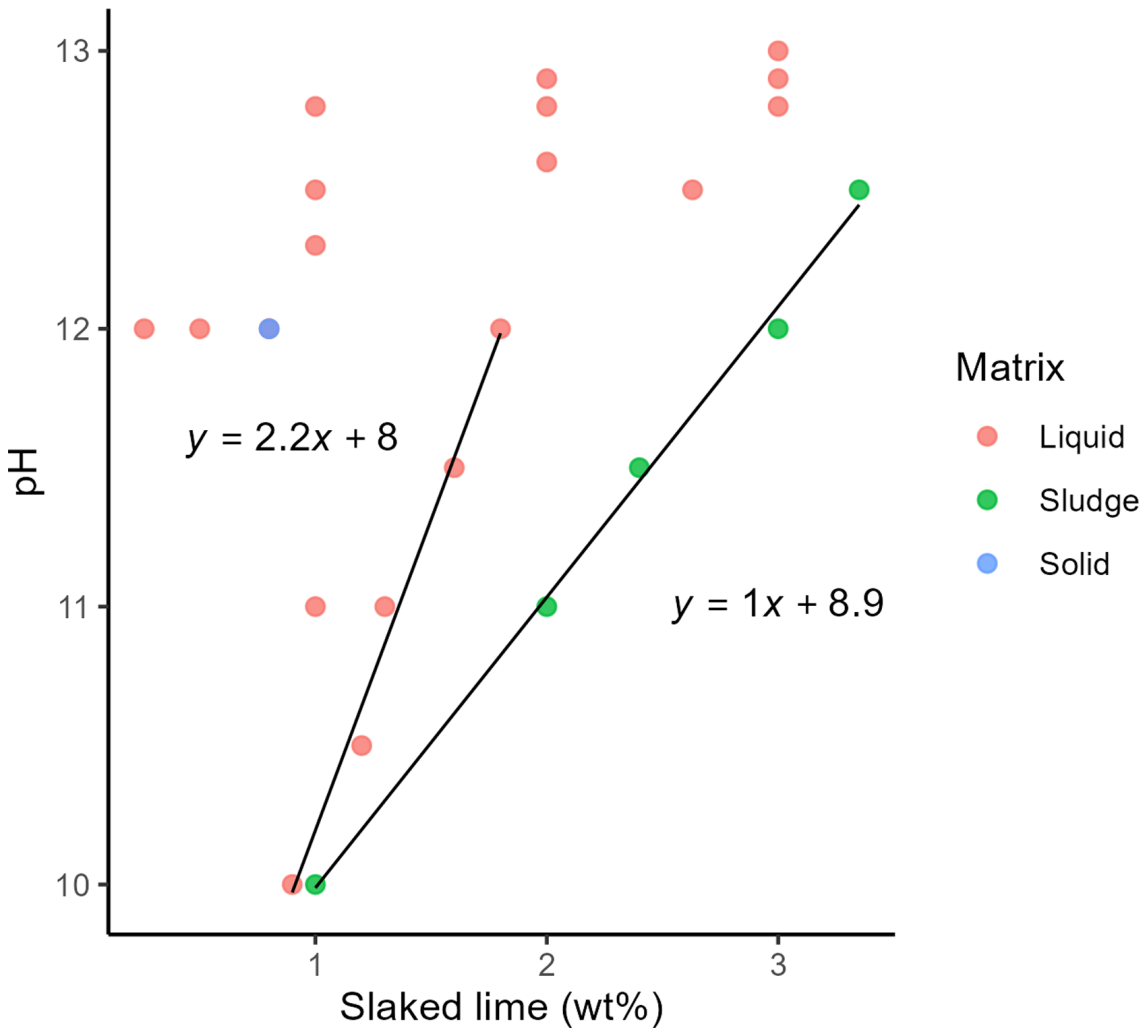
Relationship between the dosage of slaked lime (wt%) on a wet matter basis and pH after lime addition, categorized using the total suspended solids: liquid (< 12%), sludge (12–30%), and solid (> 30%).

Five additional studies were included to estimate the additive dosage required to achieve an alkaline pH (52–56). The pH values of the treated sludge and wastewater were a function of the quantity of alkali additives on a wet matter basis (wt%) (i.e., the mass of alkali additives in a 100 grammes of matrix including water) to increase the pH of the matrices (Figure 4; Supplementary Figure S4). Synthetic alkaline chemicals, including slaked lime, burnt lime, and sodium hydroxide, increased the pH of the matrix with a lower mass than ash. More data were collected for slaked lime in the liquid, sludge, and solid matrices (Supplementary Table S2); therefore, we estimated the dosage of slaked lime to increase the pH of a matrix. A higher pH was achieved with a lower lime dose in the liquid matrix (Figure 4). The pH of the sludge increased in proportion to the lime dosage. However, the increase of pH started to decrease at >2% slaked lime addition, therefore we did not include the data at pH > 12 in the further regression analysis. A linear relationship was identified between the pH values and maximum lime dosage for each pH (*p* < 0.05, nonzero slope *t*-test). Normal Q-Q plot was shown in Supplementary Figure S5.

**Figure 5:**
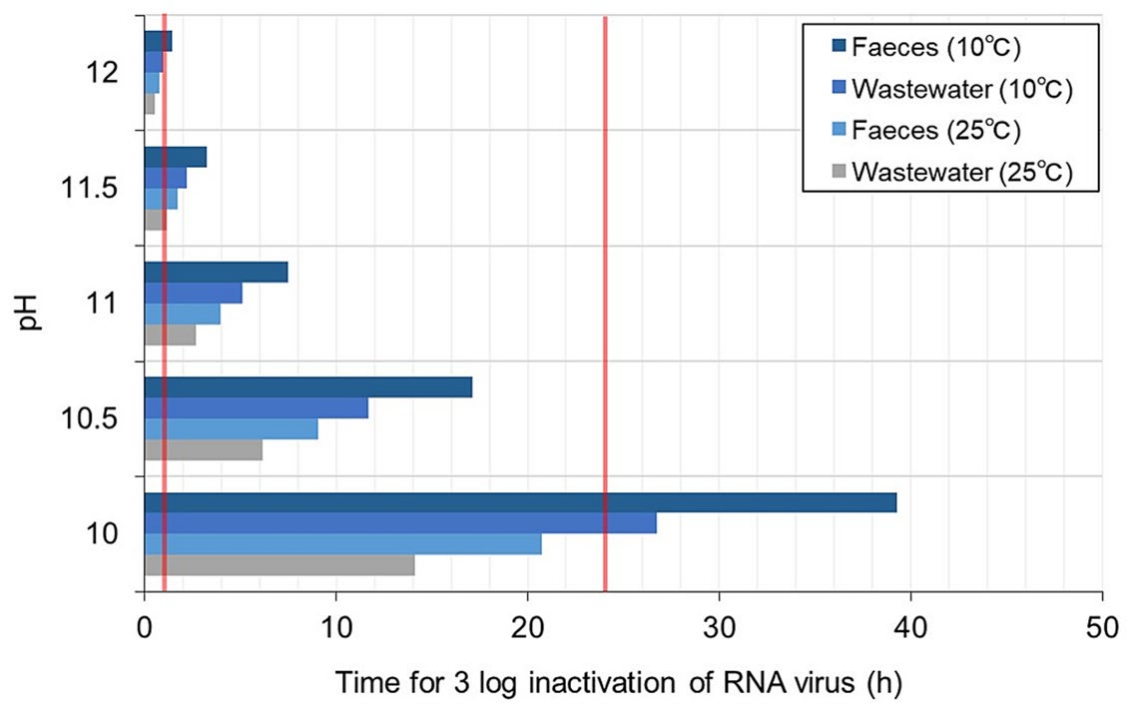
Estimated contact time for 3 log inactivation of RNA virus in wastewater (TSS <12%) and faeces (TSS = 12–30%).

The estimated T99.9 for RNA virus in different alkaline pH, temperatures, and matrices are presented in Figure 5. The T99.9 was longer for the faeces than the wastewater. At higher temperatures, T99.9 was <1 h at pH > 11.5 and exponentially prolonged at a lower pH (21 h at pH 10) for the faeces. At lower temperatures, T99.9 was <2 h at pH 12. T99.9 for the faeces was almost twice as long at the higher temperature and pH 10 (almost 40 h). The T99.9 and lime dosage required to achieve the corresponding pH are summarised in Table 2. The exponential increase in the estimated T99.9 indicated the importance of precise pH measurement and record-keeping of treatment time at pH < 11.

**Table 2:** Contact time for 3 log inactivation, pH, and dosage of slaked lime (wt%) on a wet matter basis to achieve the corresponding pH.

	pH				
	10	10.5	11	11.5	12
Wastewater					
Lime dosage (wt%)	0.9	1.2	1.4	1.6	1.8
Treatment time at 10°C (h)	26.7	11.7	5.1	2.2	1.0
Treatment time at 25°C (h)	14.1	6.2	2.7	1.2	0.5
Faeces					
Lime dosage (wt%)	1.1	1.6	2.1	2.6	3.1
Treatment time at 10°C (h)	39.3	17.1	7.5	3.3	1.4
Treatment time at 25°C (h)	20.7	9.1	4.0	1.7	0.8

In the developed model, 52 estimates were shorter than the observed data (<10 h), indicating the possible underestimation of contact time using the developed models. We carefully examined mispredicted data to avoid misleading treatment conditions (Supplementary Figure S5). More contact times for DNA phages were underestimated; the largest deviations were observed in the DNA phage estimates, and most data (28 out of 185) were mispredicted. Meanwhile, the longest underestimation of the RNA virus contact time was +15 h, which was 15.6 h at pH 11.

## 4. Discussion

This study aimed to determine the alkali dosage and contact time required for virus inactivation in faecal sludge and wastewater with high solid loading. Since alkaline treatment is widely used for sludge treatment, we identified several reviews aimed at evaluating the pathogen inactivation efficacy of alkaline treatment (e.g., (57–59)). However, previous reviews have provided little quantitative data on viruses and bacteriophage. Recent systematic reviews have estimated pathogen decay rates in sanitation-relevant matrices and determined the extent to which pathogen decay is influenced by parameters such as pH, temperature, and moisture content. They found that studies of viral inactivation by alkaline treatment are limited (16, 60, 61). A modelling study demonstrated the contribution of alkaline pH to bacteriophage LRV in sanitation-relevant matrices (61). We assumed that viral inactivation in alkaline treatment is almost determined by hydroxide ions and included studies conducted in alkaline buffer solutions or suspensions, as such we trained a model with a larger number of datasets with viral LRVs and corresponding matrix’s pH in alkaline treatment. To differentiate the outcome LRVs data according to the type of matrix (i.e., natural or synthetic), it was used as a model feature. The coefficient of matrix type was positive, indicating that a longer contact time is required for the treatment of natural matrices including wastewater and faecal sludge.

The pH of the matrix was the predominant factor causing an exponential increase in the contact time. A previous study also applied a logarithmic scale for T99 against the pH of different organisms, including phages and mammalian viruses (16). This study describes this relationship using a model that employs other factors for better T99 prediction. Hijikata et al. evaluated the first-order decay rate (inactivation rate constants) of coliphage MS2 and the compost’s pH (45). They observed a steep increase in the inactivation rate constant at pH >11, and the inactivation rate constants were described as quadratic pH functions. These findings implied that the decay of the surrogate virus was proportional to the hydrogen ion concentration.

Lime treatment is a promising measure where prolonged human waste storage is inappropriate in densely populated areas, as 1 day is sufficient to achieve a 3-log virus reduction. When matrix pH <12 because of limited alkaline additives available, the required contact time with lime should be increased considerably: T99.9 for faeces was 4.0 h at pH 11 and 20.7 h at pH 10 (25°C). Therefore, we recommend precise pH monitoring of the matrix soon after adding disinfectants.

Our results indicated that slaked lime and burnt lime increased the pH of the matrix with a lower mass than ash. A previous systematic review also reported that ash does not appear to have the same level of disinfection efficacy as lime for the treatment of faecal sludge (60). The required dosage of slaked lime estimated using the linear models was greater in the sludge than in the liquid matrix (Supplementary Figure S4). This is attributed to the greater reduction in hydroxide ions due to calcium carbonate formation from the higher organic suspended solid loading of the sludge. At least 12% slaked lime on dry matter basis (i.e., 120 g of slaked lime for a 1,000 g of dried sludge) is recommended to achieve a pH > 12 (16). We predicted that the lime dosage would be reduced by two-thirds in the liquid matrix. The addition of 3 % slaked lime on a wet basis corresponded to 10 per cent slaked lime on a dry basis for sludge with a total solids content of about 30 per cent, which is almost identical to the suggestions in a previous review (16). The estimated lime dosage required to achieve a pH of 12 was twice that required to achieve a pH of 10 for a liquid matrix, including wastewater. Meanwhile it was 2.8 times greater for the sludge matrix, including faeces. Thus, adding approximately double the quantity of slaked lime can substantially shorten the treatment time. In other words, accommodating the increased expense of adding double quantity of disinfectants could improve the disinfection efficiency.

We estimated the treatment times for RNA viruses because many waterborne viruses in excreta are RNA viruses, including Picornaviridae, Caliciviridae, Hepeviridae, Reoviridae, and Astroviridae (62). They are less fragile in the environment and are normally transmitted via the faecal-oral route. The recommended treatment conditions are as follows: <6 months at pH >11 for faecal materials (63), ≥72 h at pH 12, and 25°C for Class A biosolids, and 2 h at pH >12 for Class B biosolids (64). The estimated T99.9 at pH 12 in this study was shorter than the recommended durations, indicating that the treatment conditions in the current guidelines are sufficient for 3 log inactivation of mammalian RNA viruses. Three out of 33 observations were mispredicted in our model, and the most critical underestimation was 1.1 h versus 16 h (14.9 h shorter) as T90 at pH 12 (Supplementary Figure S6). Therefore, we propose 1-day contact time as a conservative T90.

The extent of misprediction was exceptionally large for DNA phages, indicating that the time for inactivating DNA phage should be relatively longer (Supplementary Figure S6). The waterborne DNA virus family that needs special attention is Adenoviridae (62); however, they are less tolerant in the environment compared to the DNA phages identified in this study (65–68); thus, DNA phages have been recommended as conservative surrogates in several disinfection methods, including solar disinfection and ammonia treatment. Although the inactivation time of DNA mammalian virus was shorter than that for phages in our analysis, available data on mammalian DNA viruses was limited (*n* = 5). More treatment data on mammalian DNA viruses are required to discuss the applicability of DNA phage as an appropriate surrogate in alkaline treatment of sanitation-relevant matrices. The prediction accuracy for LRVs of DNA phages was lower than for other surrogate types, indicating that the prediction of DNA phages was less reliable. This could be due to our assumption that ignored the variable tolerance between four different phages included in our dataset (i.e., coliphage ΦX174, T4, somatic coliphages, and *Bacteroides fragilis* phage). Incorporating the species differences as a feature or developing individual models may improve the prediction accuracy, while more data from experimental conditions would be needed to reduce the uncertainty due to incomplete coverage of the domain in modelling.

This study has several limitations. The virucidal effect of uncharged ammonia has been well studied. Viral inactivation by uncharged ammonia is enhanced at alkaline pH, but we did not include the concentration of uncharged ammonia as a feature because only five studies reported ammonium concentration and pH (42, 49, 51, 69, 70). A review of disinfection for sanitation-relevant matrix showed that treatment time to achieve a 2 log_10_ inactivation was shorter than for ammonia treatment. T99 ranged from 1 day to 800 days at 0.1–600 mM NH_3_, while it was within 1 day at pH 9–12 (16). Although ammonia is an important virucidal factor, we assumed that increasing pH is more important in alkaline treatment and therefore prioritised increasing the size of the dataset by looking over the effect of ammonia in modelling. Nevertheless, the prediction accuracy of the model can be improved by using ammonia concentration as a feature.

Another limitation is that the LRVs for predicting storage time were lower than the overall LRVs required for waterborne viruses in raw excreta. Reducing the pathogen count in wastewater by four LRVs is necessary to achieve a health-based target of 10^−6^ DALY per person yearly for rotavirus (71). The corresponding reduction in raw faecal material is six LRVs (63). Only a few studies have reported such high LRVs (47, 49, 69). Due to the limitations of the available datasets, a model for the time required for the six LRVs was not constructed. Extrapolation of LRVs in the developed model is not recommended because the time for larger LRVs is not the sum of the times for smaller LRVs when viral decay does not follow first-order kinetics, as in previous studies (17, 36, 42). Larger LRV estimates should be validated via disinfection tests using surrogate viruses with higher infectivity titers in the initial faecal matter or wastewater. More importantly, the risk level depends on several unknown factors, including the concentration of infectious viruses in wastewater, viral infection dose, and the likelihood of transmission via faecal materials. Larger LRVs are not required when a virus is excreted in a smaller number (<10^4^) in faeces or when additional risk barriers (e.g., disinfection of the environmental surface and drinking water) are introduced in the faecal-oral route. Anaerobic treatments including anaerobic baffled reactor, anaerobic filter, and biogas reactor are applicable onsite, while more treatments are used as semi-centralized treatment (e.g., drying bed) (72).

## 5. Conclusion

We conducted a systematic review to determine the contact time with alkali additives for treating a virus-containing matrix. Fourteen studies reported quantitative information on their disinfection tests, including the time-series decay of viruses, types of matrices, and physicochemical properties. We obtained synthesised datasets of the required contact time associate to pH, temperature, matrix type, virus type, and initial concentration of virus. The size of datasets applied to further analysis was 136 for T90, 105 for T99, 77 for T99.9, and 57 for T99.99. The pH of the matrix and contact time with lime were determined using machine learning algorithms. The slaked lime quantity required to achieve pH 12 was 1.8 and 3.1% on the wet matter basis of wastewater and faeces, respectively. When matrix pH <12 because of limited alkaline additives available, the required contact time with lime should be increased considerably: T99.9 for faeces was 4.0 h at pH 11 and 20.7 h at pH 10 (25°C). Thus, lime treatment is a promising measure where prolonged human waste storage is inappropriate in densely populated areas, as 1 day is sufficient to inactivate viruses. Nevertheless, further studies are necessary to investigate the contact time for >3 log inactivation of RNA virus.

## Data availability statement

The original contributions presented in the study are included in the article/Supplementary material, further inquiries can be directed to the corresponding author.

## Author contributions

WO: Conceptualization, Data curation, Funding acquisition, Investigation, Methodology, Writing – original draft. DS: Data curation, Supervision, Writing – review & editing.
