# Supplementary material for: Estimation of alkali dosage and contact time for treating human excreta containing viruses as an emergency response: a systematic review
Source: Front Public Health. 2023 Nov 10;11:1286595. doi: 10.3389/fpubh.2023.1286595 (PMC10667465; doi:10.3389/fpubh.2023.1286595)
Supplement: Supplementary file 1 [file Data_Sheet_1.docx]

Supplementary Material

# R code for the estimation of Hom’s model parameters

d<-data.frame(t,N) # t: time, N: virus concentration

param0 <- c(7,0.1,.00001) # Initial setting of *k* and *m*

#optim

mlem <- function(p, data) {

parameters<-c(k=p[1], m=p[2], sigma2=p[3])

rss<-data[1,2]*exp(-p[1]*data[,1]^p[2])

n<-length(data)

sum(-log(data[,2]))-(n/2)*log(2*pi*parameters[3])-1/2/parameters[3]*sum((log(rss)-log(data[,2]))^2)

}

fit0 <- optim(param0, mlem, data=d, control=list(fnscale=-1))

fit0$par

fit0$convergence # Check the convergence

write.table(round(fit0$par[1:2],digits=4), file="clipboard", sep="\t", row.names=FALSE, col.names=FALSE)

plot(data.frame(t,-fit0$par[1]*t^fit0$par[2])) # Visualize

# Python code for the ARD (Modeling T99.9 in this example)

import pandas as pd

import numpy as np

df=pd.read_csv("lime_virus.csv", sep=",")

df2=df[["Phage", "RNA", "T", "pH", "lgN0", "Cmpx","Sticky","t3"]]

df2=df2.dropna(how='any') # Delete row containing NaN

X=df2[["Phage", "RNA", "T", "pH", "lgN0", "Cmpx","Sticky"]]

y=np.log10(df2["t3"])

# Create a csv file to output ten MSEs in each cross-validation

import csv

outfile = open('limeT999_ARD.csv','w', newline='')

writer = csv.writer(outfile)

writer.writerow(['j','ard_MSE_train', 'ard_MSE_test',"ard_MAE_train","ard_MAE_test",'ardR2_train','ardR2_test'])

for j in range(10):

X_train, X_test, y_train, y_test = train_test_split(X, y, test_size=0.2, random_state=j )

np.savetxt('T999_train_X.csv', X_train, delimiter=',')

np.savetxt('T999_test_X.csv', X_test, delimiter=',')

np.savetxt('T999_train_Y.csv',y_train, delimiter=',')

np.savetxt('T999_test_Y.csv', y_test, delimiter=',')

####Standard Scaler####

from sklearn.preprocessing import StandardScaler

sc = StandardScaler()

scaler = sc.fit(X_train)

X_train=scaler.transform(X_train)

X_test=scaler.transform(X_test)

########################

####k-fold-cross-validation####

########################

####Grid search for Regularized Regressions####

## k-Fold cross validation and grid search ##

from sklearn.model_selection import GridSearchCV

from sklearn import linear_model

###########

###

param_ard={'alpha_1':10**np.arange(-6,-4,0.5),

'alpha_2':10**np.arange(-3,0,0.5),

'lambda_1':10**np.arange(-2,0,0.2),

'lambda_2':10**np.arange(-3,0,0.2)}

ARD=linear_model.ARDRegression()

ard=GridSearchCV(ARD, param_ard, cv=5)

ard.fit(X_train, y_train)

ard_pred_test=ard.predict(X_test)

ard_pred_train=ard.predict(X_train)

# Score #

from sklearn.metrics import mean_squared_error, mean_absolute_error

ard_mse_test=mean_squared_error(y_test, ard_pred_test)

ard_mse_train=mean_squared_error(y_train, ard_pred_train)

ard_mae_test=mean_absolute_error(y_test, ard_pred_test)

ard_mae_train=mean_absolute_error(y_train, ard_pred_train)

ardR2_train=ard.score(X_train, y_train)

ardR2_test=ard.score(X_test, y_test)

####Model Information####

# Best hyperparameters

print("Best params ARD: {}".format(ard.best_params_))

writer.writerow([j, ard_mse_train, ard_mse_test, ard_mae_train, ard_mae_test, ardR2_train, ardR2_test])

outfile.close()

#########　Train model using all the data set

from sklearn.preprocessing import StandardScaler

scaler=StandardScaler()

scaler.fit(X)

X=scaler.transform(X)

################

from sklearn import linear_model

from sklearn.model_selection import GridSearchCV

from sklearn.metrics import mean_squared_error, mean_absolute_error

param={'alpha_1':10**np.arange(-6,1,0.5), # Change the hyperparameter values

'alpha_2':10**np.arange(-6,1,0.5),

'lambda_1':10**np.arange(-4,1,0.5),

'lambda_2':10**np.arange(-4,1,0.5)}

ard=linear_model.ARDRegression()

clf_ard=GridSearchCV(ard, param, cv=5)

clf_ard.fit(X,y)

clf_pred=clf_ard.predict(X)

clf_ard_MSE=mean_squared_error(y, clf_pred)

print("mse:{}".format(clf_ard_MSE))

print("rmse:{}".format(np.sqrt(clf_ard_MSE)))

print("R2_bestmodel:{}".format(clf_ard.score(X_test,y_test)))

print("Best params: {}".format(clf_ard.best_params_))

ARD_best=linear_model.ARDRegression(**clf_ard.best_params_)

ARD_best.fit(X, y)

ARD_pred=ARD_best.predict(X)

ARD_MSE=mean_squared_error(y, ARD_pred)

print("mse_test_bestmodel:{}".format(ARD_MSE))

Pred_Tr=ARD_best.predict(X_train)

print("MSE_Tr:{}".format(mean_squared_error(y_train, Pred_Tr)))

print("RMSE_Tr:{}".format(np.sqrt(mean_squared_error(y_train, Pred_Tr))))

print("R2_Tr:{}".format(ARD_best.score(X_train,y_train)))

# Prediction

df_sim=pd.read_csv('lime_virus_pre_230314.csv')

X2=df_sim[["Phage", "RNA", "T", "pH", "lgN0", "Cmpx","Sticky"]]

scaler2=StandardScaler()

scaler2.fit(X2)

X_sim=scaler.transform(X2)

Pred=ARD_best.predict(X_sim)

# Figures and Tables


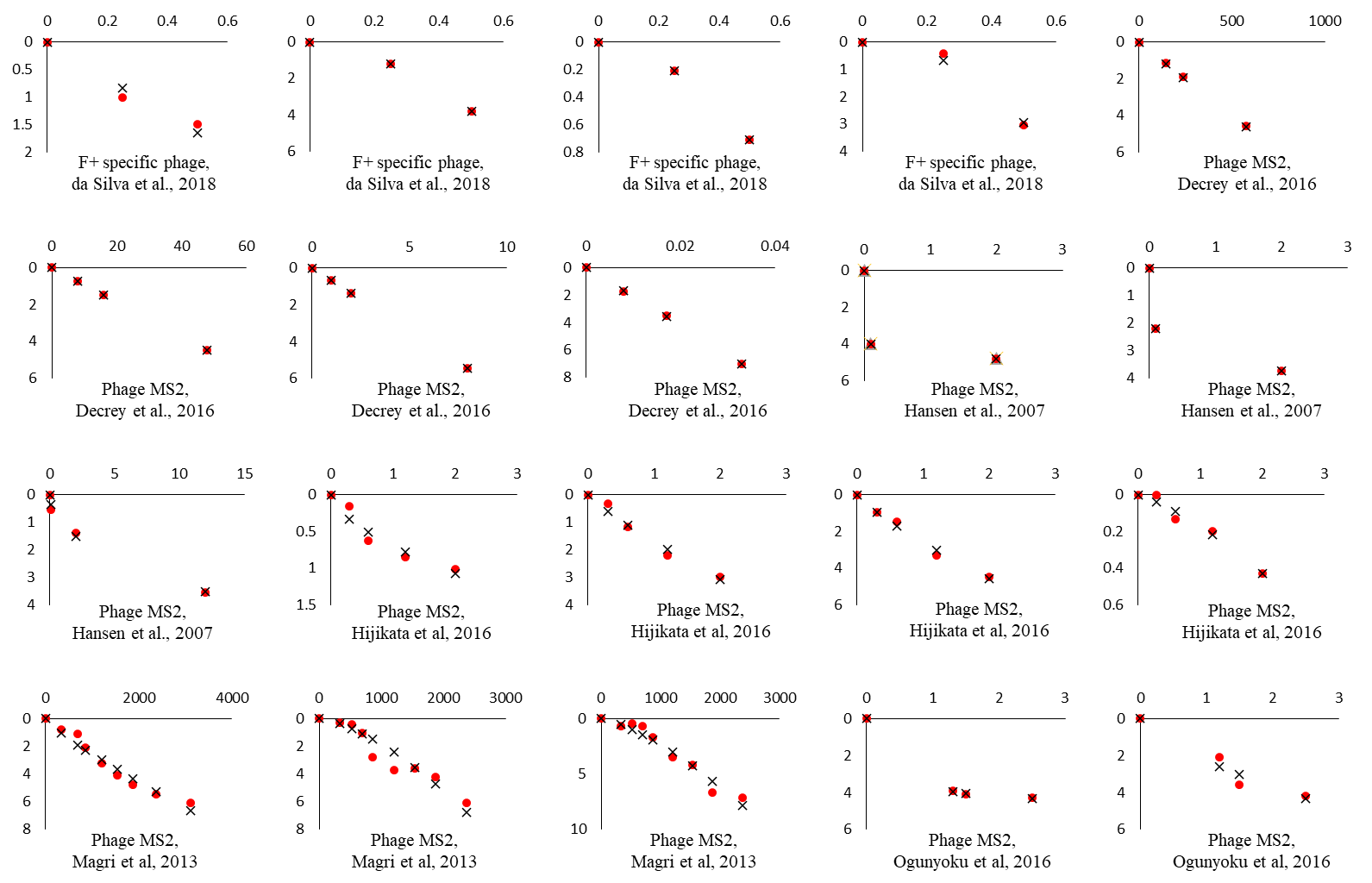


Figure S1. Time (hourly) course decay of log reduction values (LRV). Crosses represent LRVs predicted by Hom’s model, while circles represent LRVs observed in experimental studies (continued).


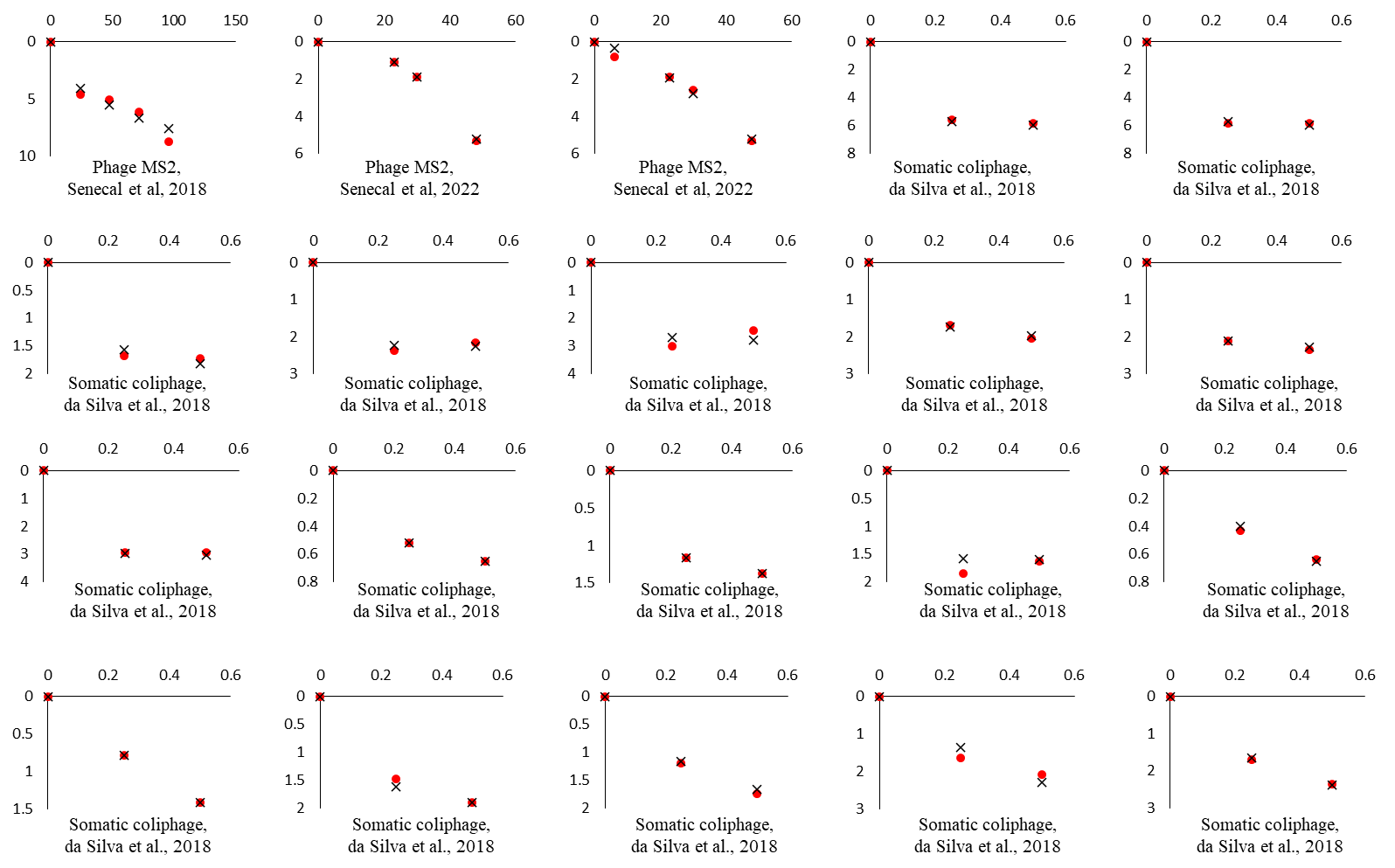


Figure S1. Time (hourly) course decay of log reduction values (LRV). Crosses represent LRVs predicted by Hom’s model, while circles represent LRVs observed in experimental studies (continued).


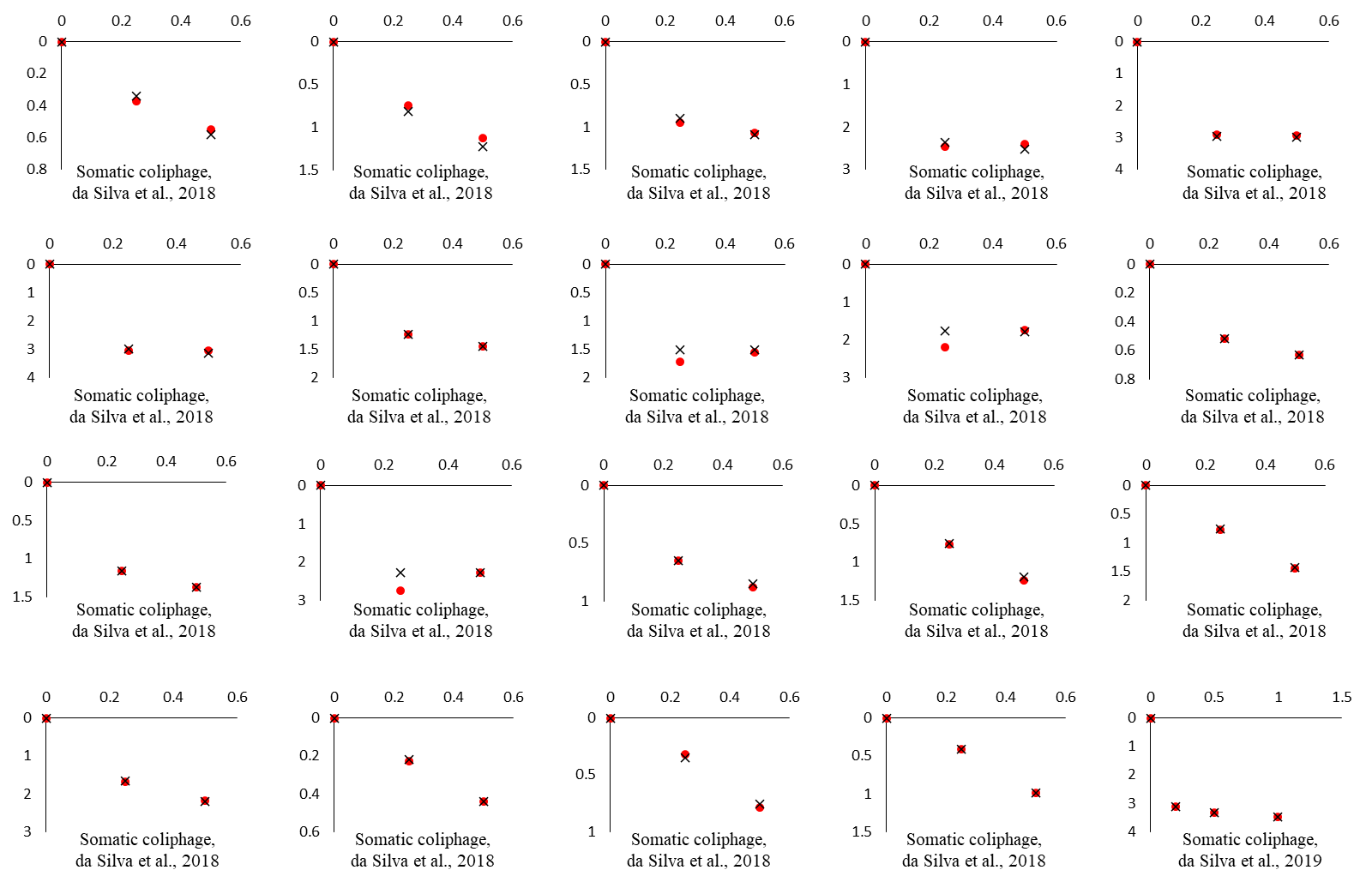


Figure S1. Time (hourly) course decay of log reduction values (LRV). Crosses represent LRVs predicted by Hom’s model, while circles represent LRVs observed in experimental studies (continued).


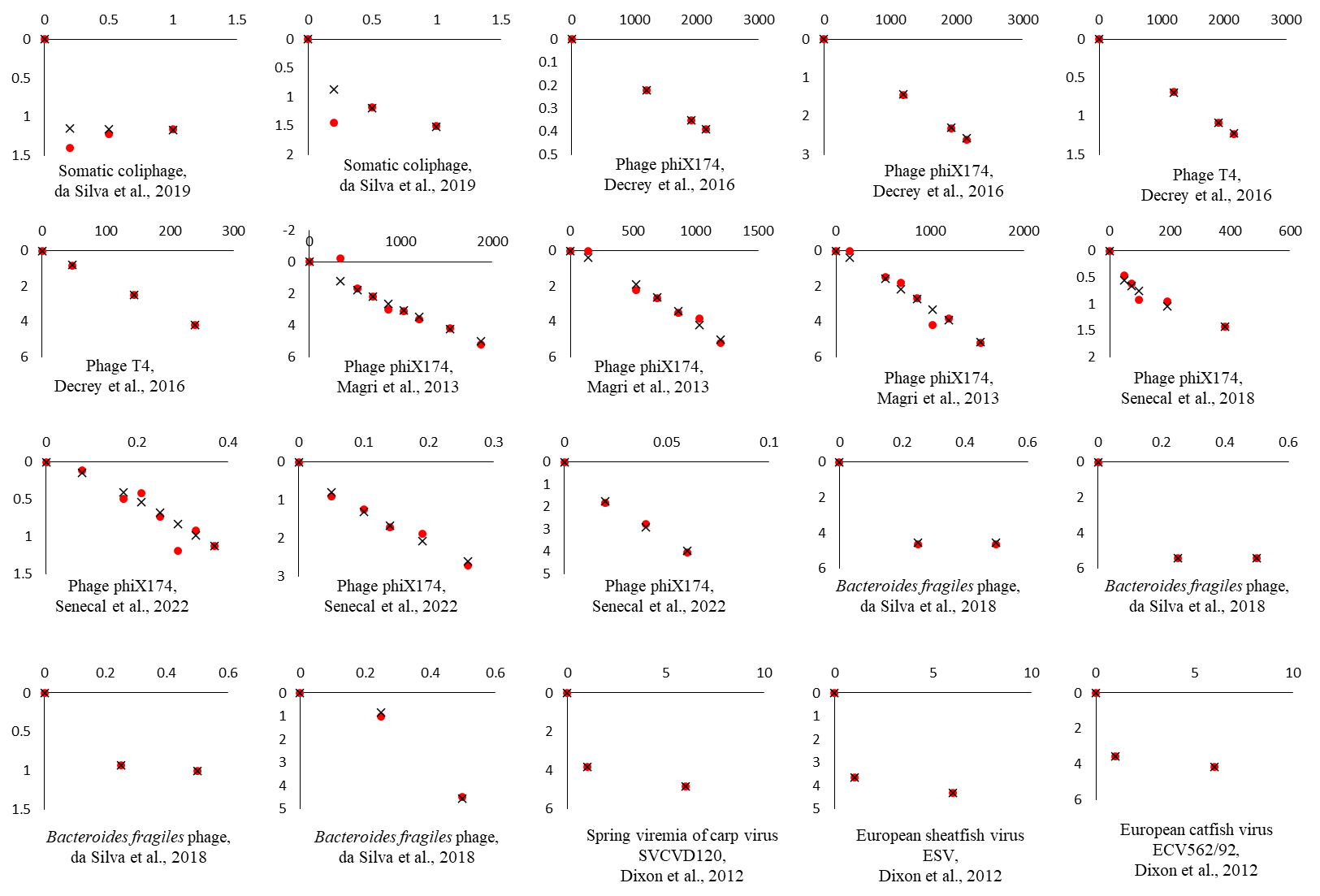


Figure S1. Time (hourly) course decay of log reduction values (LRV). Crosses represent LRVs predicted by Hom’s model, while circles represent LRVs observed in experimental studies (continued).


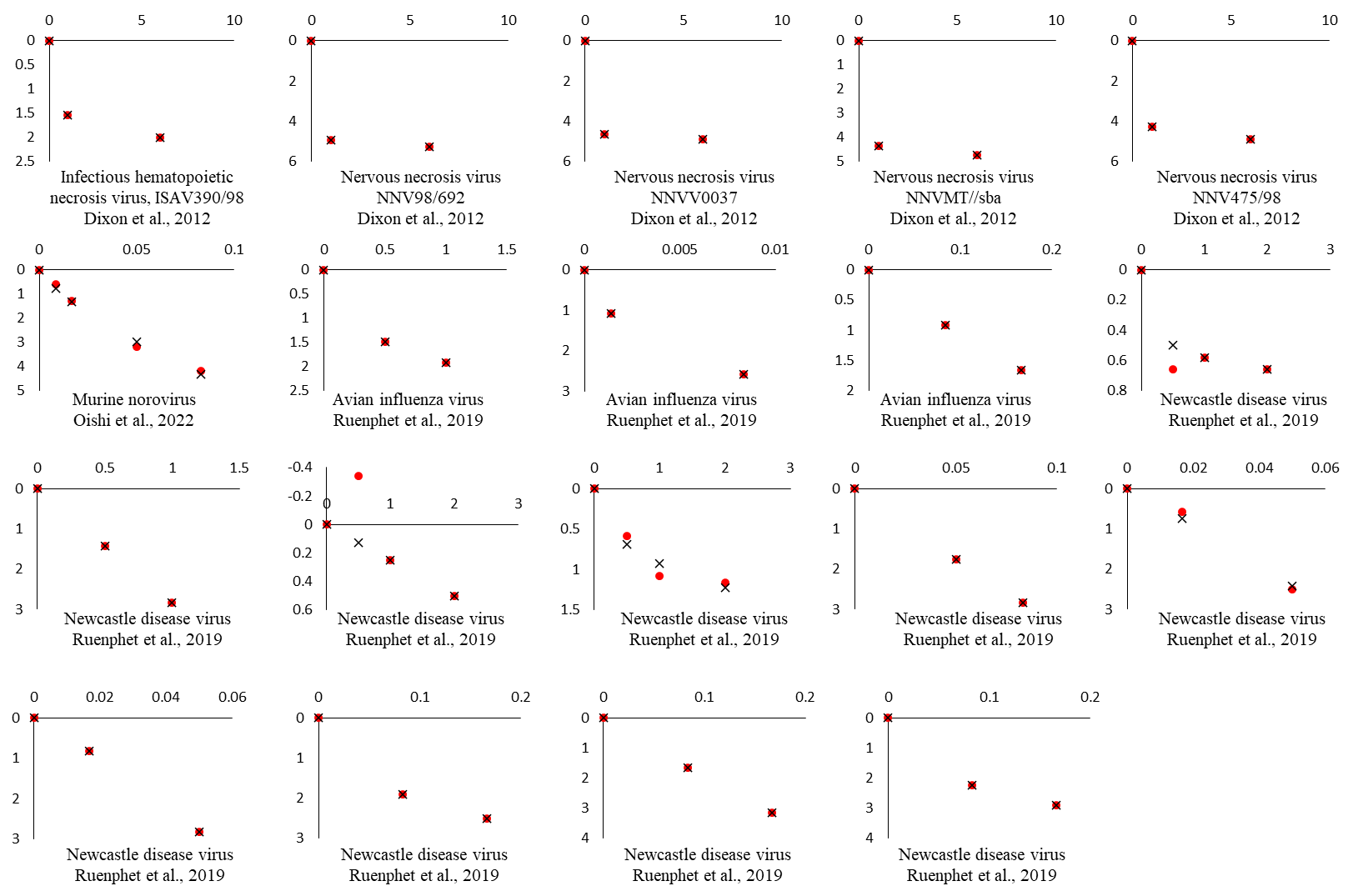


Figure S1. Time course (hourly) of log reduction values (LRVs). Crosses represent LRVs predicted by Hom’s model, while circles represent LRVs observed in experimental studies.


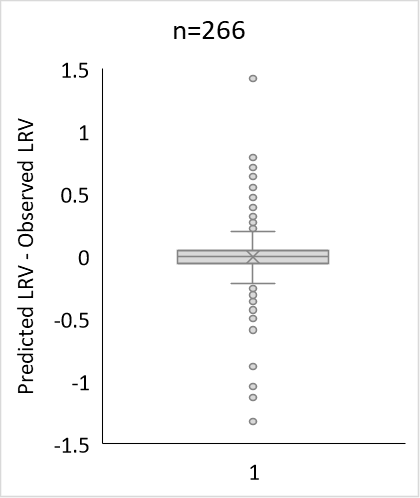


Figure S2. Deviations between predicted log reduction values (LRVs) from Hom’s model and observed LRVs.


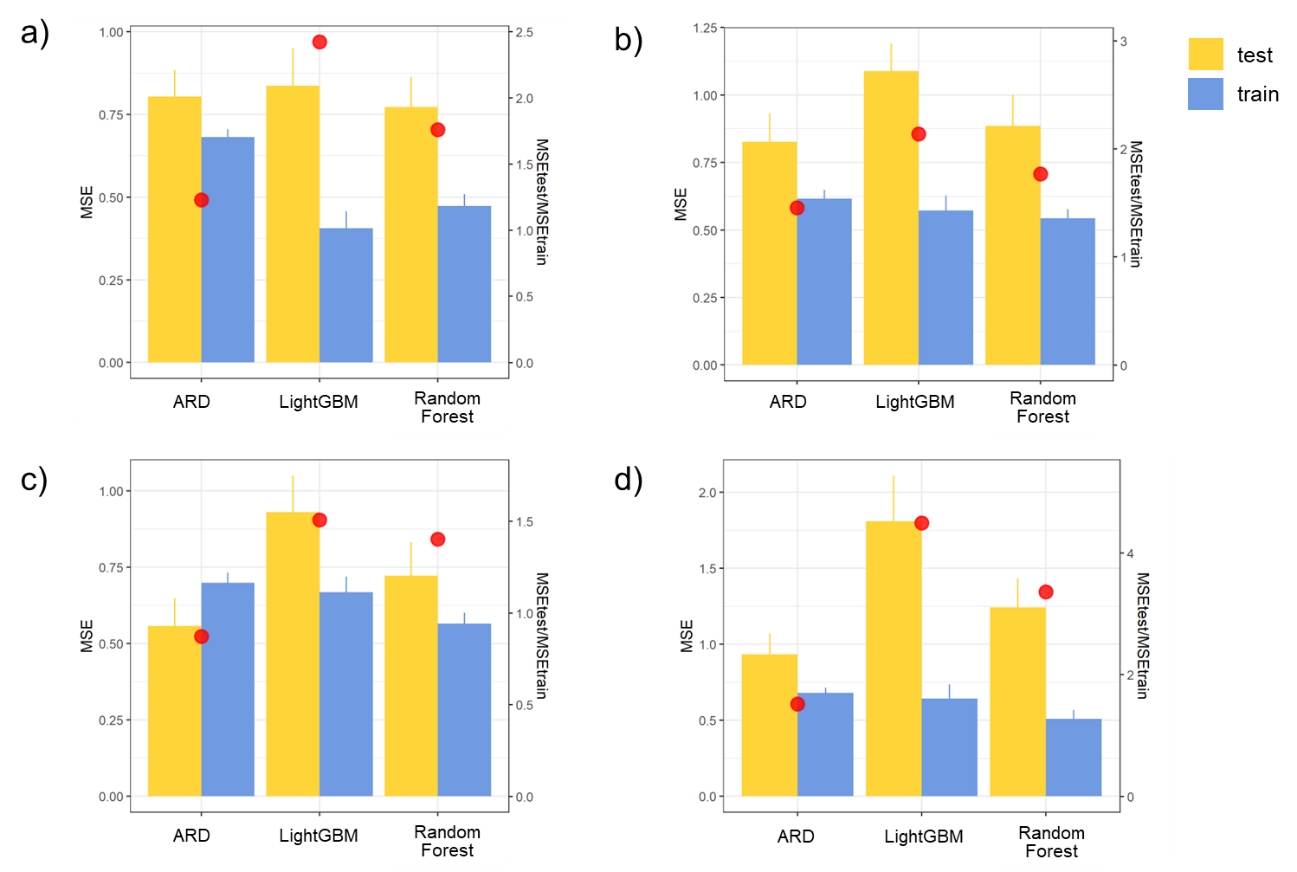


Figure S3. Mean value of mean squared error in prediction of training data (MSEtrain) and test data (MSEtest) by the three models for a) T90, b) T99, c) T99.9, and d) T99.99. Red plots represent the ratio of MSE values indicative for overfitting to training data.


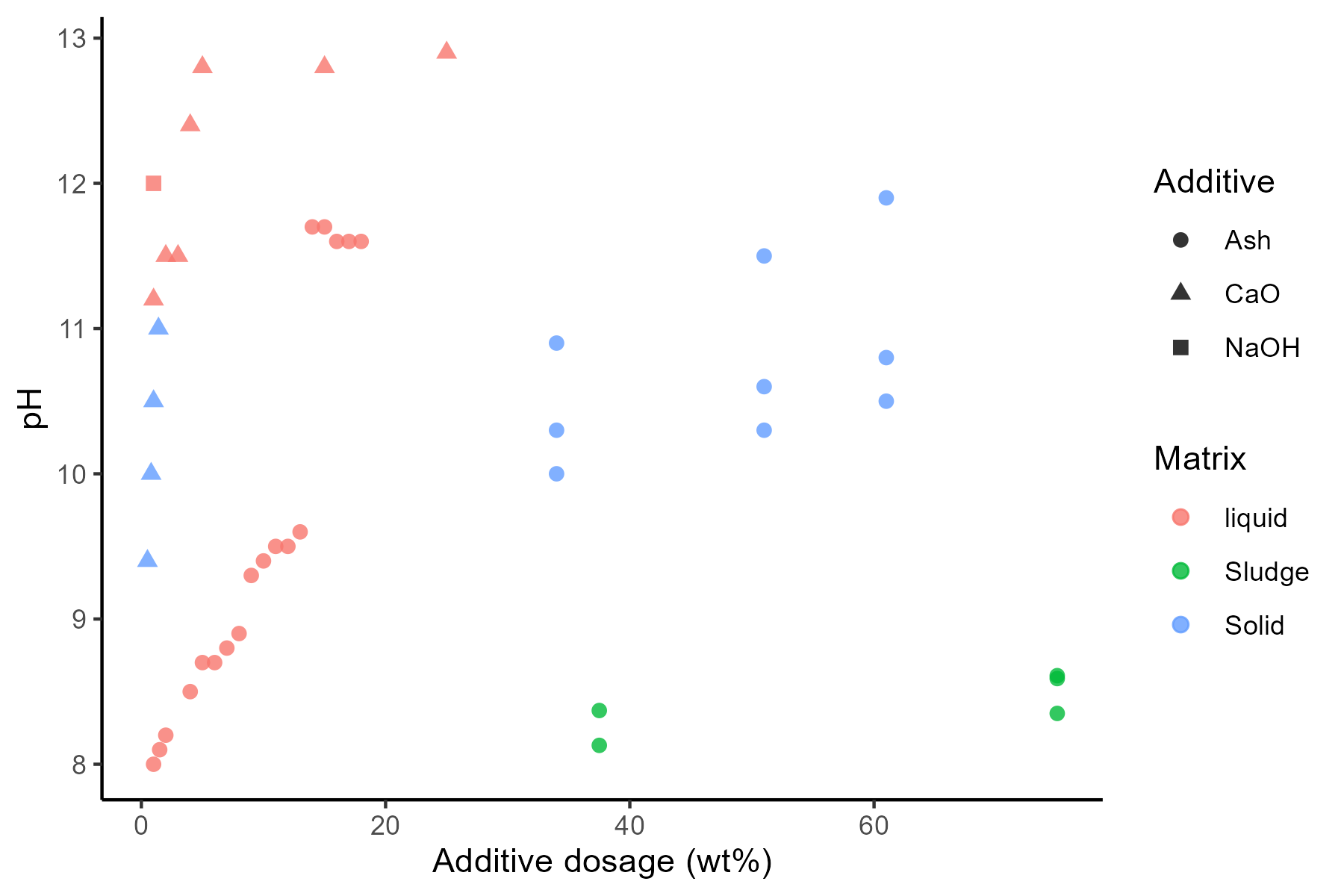


Figure S4. Relationship between the dosage of additive (wt%) on wet matter basis and pH after the addition, which was categorized by total suspended solids: liquid (< 12%), sludge (12-30%), and solid (> 30%).

a)


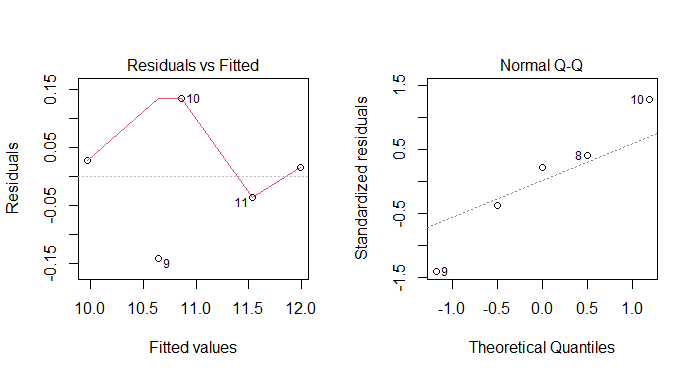


b)


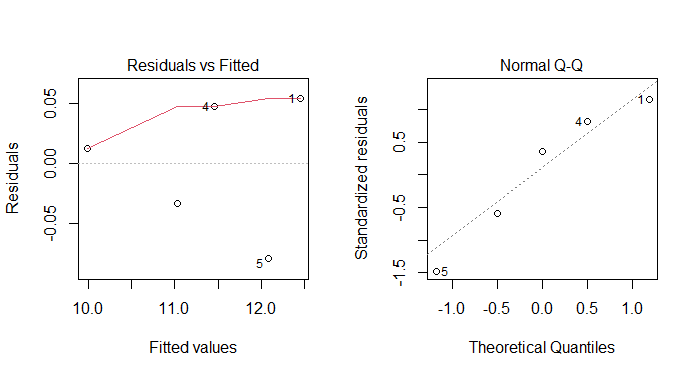


Figure S5. Normal Q-Q plot for regression analysis: a) for a liquid matrix and b) for a sludge matrix.


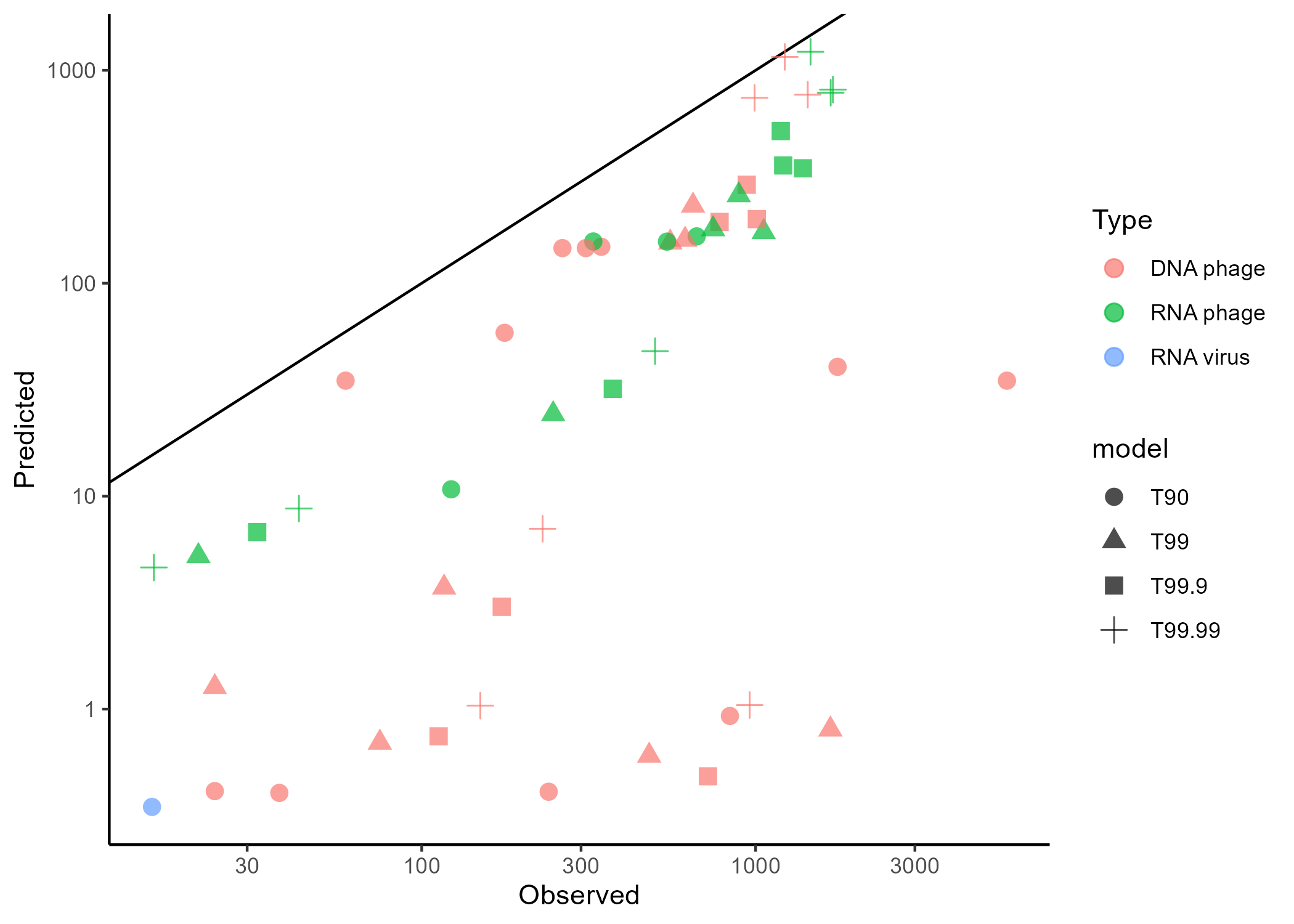


Figure S6. Time for inactivation which were underestimated > 10 h by the better models.

Table S1. Dataset used for the modeling. T is temperature, and lgN0 is initial concentration of surrogate. Dummy variables are: Phage (1: phage, 0: virus), RNA (1: RNA, 0: DNA), Cmpx (1: natural matrix, 0: synthesized matrix), Sticky (0: liquid matrix, 1: sludge, 2: solid).

| Paper | Virus | Phage | RNA | T | pH | lgN0 | Cmpx | Sticky | t1 | t2 | t3 | t4 | Time(hr) | LRV |
| --- | --- | --- | --- | --- | --- | --- | --- | --- | --- | --- | --- | --- | --- | --- |
| Bean et al., 2017 | MS2 | 1 | 1 | 28 | 12 | 6.22 | 0 | 0 | 1 | 1 | 1 | 1 | 0 | 0 |
|  |  |  |  |  |  |  |  |  |  |  |  |  | 1 | 4.48 |
|  | HAdV-5 | 0 | 0 | 28 | 12 | 4.47 | 0 | 0 | 0.1 | 0.1 | 0.1 | 0.1 | 0 | 0 |
|  |  |  |  |  |  |  |  |  |  |  |  |  | 0.1 | >4 |
|  | RV-WA | 0 | 1 | 28 | 12 | 4.09 | 0 | 0 | 0.1 | 0.1 | 0.1 | 0.1 | 0 | 0 |
|  |  |  |  |  |  |  |  |  |  |  |  |  | 0.1 | >4 |
| da Silva et al., 2018 | SOMPH | 1 | 0 | 21 | 12.9 | 5.63 | 1 | 0 | 0.5 | 0.5 | 0.5 | 0.5 | 0 | 0 |
|  |  |  |  |  |  | 0 |  |  |  |  |  |  | 0.5 | 5.63 |
|  | SOMPH | 1 | 0 | 21 | 13.4 | 5.86 | 1 | 0 | 0.25 | 0.25 | 0.25 | 0.25 | 0 | 0 |
|  |  |  |  |  |  | 0 |  |  |  |  |  |  | 0.25 | 5.86 |
|  | SOMPH | 1 | 0 | 21 | 12.91 | 5.63 | 1 | 0 | 0.5 | 0.5 | 0.5 | 0.5 | 0 | 0 |
|  |  |  |  |  |  | 0 |  |  |  |  |  |  | 0.5 | 5.63 |
|  | F+PH | 1 | 1 | 21 | 12.9 | 4.34 | 1 | 0 | 0.25 |  |  |  | 0 | 0 |
|  |  |  |  |  |  | 2.58 |  |  |  |  |  |  | 0.25 | 1.76 |
|  | GB124PH | 1 | 0 | 21 | 12.8 | 6.08 | 1 | 0 | 0.25 | 0.25 | 0.25 | 0.25 | 0 | 0 |
|  |  |  |  |  |  | 1 |  |  |  |  |  |  | 0.25 | 5.08 |
|  | GB124PH | 1 | 0 | 21 | 12.93 | 6.08 | 1 | 0 | 0.25 | 0.25 | 0.25 | 0.25 | 0 | 0 |
|  |  |  |  |  |  | 1 |  |  |  |  |  |  | 0.25 | 5.08 |
|  | GB124PH | 1 | 0 | 21 | 13.02 | 6.08 | 1 | 0 | 0.25 | 0.25 | 0.25 | 0.25 | 0 | 0 |
|  |  |  |  |  |  | 1.3 |  |  |  |  |  |  | 0.25 | 4.78 |
|  | GB124PH | 1 | 0 | 21 | 12.52 | 5.78 | 1 | 0 | 0.25 | 0.25 | 0.25 | 0.25 | 0 | 0 |
|  |  |  |  |  |  | 0.7 |  |  |  |  |  |  | 0.25 | 5.08 |
|  | GB124PH | 1 | 0 | 21 | 12.72 | 5.78 | 1 | 0 | 0.25 | 0.25 | 0.25 | 0.25 | 0 | 0 |
|  |  |  |  |  |  | 0.7 |  |  |  |  |  |  | 0.25 | 5.08 |
|  | GB124PH | 1 | 0 | 21 | 12.75 | 5.78 | 1 | 0 | 0.25 | 0.25 | 0.25 | 0.25 | 0 | 0 |
|  |  |  |  |  |  | 1 |  |  |  |  |  |  | 0.25 | 4.78 |
|  | GB124PH | 1 | 0 | 21 | 12.91 | 5.63 | 1 | 0 | 0.25 | 0.25 | 0.25 | 0.25 | 0 | 0 |
|  |  |  |  |  |  | 0.7 |  |  |  |  |  |  | 0.25 | 4.93 |
|  | GB124PH | 1 | 0 | 21 | 12.97 | 5.63 | 1 | 0 | 0.25 | 0.25 | 0.25 | 0.25 | 0 | 0 |
|  |  |  |  |  |  | 1 |  |  |  |  |  |  | 0.25 | 4.63 |
|  | GB124PH | 1 | 0 | 21 | 12.73 | 6.11 | 1 | 0 | 0.25 | 0.25 | 0.25 | 0.25 | 0 | 0 |
|  |  |  |  |  |  | 0.48 |  |  |  |  |  |  | 0.25 | 5.63 |
|  | GB124PH | 1 | 0 | 21 | 12.95 | 6.11 | 1 | 0 | 0.25 | 0.25 | 0.25 | 0.25 | 0 | 0 |
|  |  |  |  |  |  | 0.7 |  |  |  |  |  |  | 0.25 | 5.41 |
|  | GB124PH | 1 | 0 | 21 | 12.9 | 5.78 | 1 | 0 | 0.25 |  |  |  | 0 | 0 |
|  |  |  |  |  |  | 4.38 |  |  |  |  |  |  | 0.25 | 1.4 |
| Decrey et al., 2016 | T4 | 1 | 0 | 20 | 11.5 | 6 | 0 | 0 | 0.01 | 0.01 | 0.01 |  | 0 | 0 |
|  |  |  |  |  |  | 2.65 |  |  |  |  |  |  | 0.008333 | 3.35 |
| Dixon et al., 2012 | SVCV880062 | 0 | 1 | 4 | 12 | 6.66 | 0 | 0 | 1 | 1 | 1 | 1 | 0 | 0 |
|  |  |  |  |  |  |  |  |  |  |  |  |  | 1 | 4.65 |
|  | VHSVDK3592B | 0 | 1 | 4 | 12 | 6.33 | 0 | 0 | 1 | 1 |  |  | 0 | 0 |
|  |  |  |  |  |  |  |  |  |  |  |  |  | 1 | 2.72 |
|  | VHSVIp49 | 0 | 1 | 4 | 12 | 8.34 | 0 | 0 | 1 | 1 |  |  | 0 | 0 |
|  |  |  |  |  |  |  |  |  |  |  |  |  | 1 | 2.12 |
| Hansen et al. 2007 | HAdV-5 | 0 | 0 | 22 | 12 | 6.38 | 1 | 2 | 0.1 | 0.1 | 0.1 |  | 0 | 0 |
|  |  |  |  |  |  | 2.54 |  |  |  |  |  |  | 0.1 | 3.84 |
|  | HAdV-5 | 0 | 0 | 4 | 12 | 4.58 | 1 | 2 | 0.1 | 0.1 | 0.1 |  | 0 | 0 |
|  |  |  |  |  |  | 0.95 |  |  |  |  |  |  | 0.1 | 3.63 |
|  | MS2 | 1 | 1 | 22 | 12 | 4.44 | 0 | 0 | 0.1 | 0.1 |  |  | 0 | 0 |
|  |  |  |  |  |  | 2.42 |  |  |  |  |  |  | 0.1 | 2.02 |
| Ruenphet et al., 2019 | AIV | 0 | 1 | 28 | 12.5 | 6.75 | 0 | 0 | 0.17 |  |  |  | 0 | 0 |
|  |  |  |  |  |  | 5.42 |  |  |  |  |  |  | 0.167 | 1.33 |
|  | AIV | 0 | 1 | 28 | 13 | 7.5 | 0 | 0 | 0 |  |  |  | 0 | 0 |
|  |  |  |  |  |  | 6.33 |  |  |  |  |  |  | 0.00139 | 1.17 |
|  | AIV | 0 | 1 | 28 | 12.5 | 7.58 | 0 | 0 | 0.08 |  |  |  | 0 | 0 |
|  |  |  |  |  |  | 6.25 |  |  |  |  |  |  | 0.0833 | 1.33 |
|  | AIV | 0 | 1 | 28 | 12.5 | 7.17 | 0 | 0 | 0.08 |  |  |  | 0 | 0 |
|  |  |  |  |  |  | 5.33 |  |  |  |  |  |  | 0.0833 | 1.84 |
|  | AIV | 0 | 1 | 28 | 12.5 | 7.42 | 0 | 0 | 0.17 | 0.17 |  |  | 0 | 0 |
|  |  |  |  |  |  | 5.42 |  |  |  |  |  |  | 0.1667 | 2 |
|  | AIV | 0 | 1 | 28 | 12.5 | 7.33 | 0 | 0 | 0.17 |  |  |  | 0 | 0 |
|  |  |  |  |  |  | 5.58 |  |  |  |  |  |  | 0.1667 | 1.75 |
|  | AIV | 0 | 1 | 28 | 12.5 | 7.58 | 0 | 0 | 0.17 | 0.17 |  |  | 0 | 0 |
|  |  |  |  |  |  | 5.42 |  |  |  |  |  |  | 0.1667 | 2.16 |
|  | NDV | 0 | 1 | 28 | 13 | 7.67 | 0 | 0 | 0.05 | 0.05 |  |  | 0 | 0 |
|  |  |  |  |  |  | 5.25 |  |  |  |  |  |  | 0.05 | 2.42 |
| Decrey et al., 2016 | MS2 | 1 | 1 | 20 | 10 | 10 | 0 | 0 | 122.56 | 247.54 | 373.45 | 499.97 | 0 | 0 |
|  |  |  |  |  |  | 8.85 |  |  |  |  |  |  | 144 | 1.15 |
|  |  |  |  |  |  | 8.09 |  |  |  |  |  |  | 240 | 1.91 |
|  |  |  |  |  |  | 5.41 |  |  |  |  |  |  | 576 | 4.59 |
|  | MS2 | 1 | 1 | 20 | 11 | 10 | 0 | 0 | 10.72 | 21.43 | 32.15 | 42.87 | 0 | 0 |
|  |  |  |  |  |  | 9.25 |  |  |  |  |  |  | 8 | 0.75 |
|  |  |  |  |  |  | 8.51 |  |  |  |  |  |  | 16 | 1.49 |
|  |  |  |  |  |  | 5.52 |  |  |  |  |  |  | 48 | 4.48 |
|  | MS2 | 1 | 1 | 20 | 11.5 | 10 | 0 | 0 | 1.46 | 2.93 | 4.39 | 5.85 | 0 | 0 |
|  |  |  |  |  |  | 9.32 |  |  |  |  |  |  | 1 | 0.68 |
|  |  |  |  |  |  | 8.63 |  |  |  |  |  |  | 2 | 1.37 |
|  |  |  |  |  |  | 4.53 |  |  |  |  |  |  | 8 | 5.47 |
|  | MS2 | 1 | 1 | 20 | 12 | 10 | 0 | 0 | 0 | 0.01 | 0.01 | 0.02 | 0 | 0 |
|  |  |  |  |  |  | 8.25 |  |  |  |  |  |  | 0.008 | 1.75 |
|  |  |  |  |  |  | 6.5 |  |  |  |  |  |  | 0.017 | 3.5 |
|  |  |  |  |  |  | 2.99 |  |  |  |  |  |  | 0.033 | 7.01 |
| Hansen et al. 2007 | MS2 | 1 | 1 | 22 | 12 | 6.82 | 1 | 2 | 0.1 | 0.1 | 0.1 | 0.1 | 0 | 0 |
|  |  |  |  |  |  | 2.81 |  |  |  |  |  |  | 0.1 | 4.01 |
|  |  |  |  |  |  | 2.03 |  |  |  |  |  |  | 2 | 4.79 |
|  | MS2 | 1 | 1 | 22 | 12 | 7 | 1 | 2 | 0.1 | 0.1 |  |  | 0 | 0 |
|  |  |  |  |  |  | 4.4 |  |  |  |  |  |  | 0.1 | 2.6 |
|  | MS2 | 1 | 1 | 22 | 12 | 4.44 | 0 | 0 | 0.1 | 0.1 |  |  | 0 | 0 |
|  |  |  |  |  |  | 2.42 |  |  |  |  |  |  | 0.1 | 2.02 |
|  | MS2 | 1 | 1 | 22 | 12 | 4.67 | 1 | 0 | 0.1 | 0.1 | 0.59 | 3.03 | 0 | 0 |
|  |  |  |  |  |  | 2.47 |  |  |  |  |  |  | 0.1 | 2.2 |
|  |  |  |  |  |  | 0.95 |  |  |  |  |  |  | 2 | 3.72 |
|  | MS2 | 1 | 1 | 4 | 12 | 4.66 | 1 | 0 | 0.84 | 3.64 | 8.58 | 15.77 | 0 | 0 |
|  |  |  |  |  |  | 4.1 |  |  |  |  |  |  | 0.1 | 0.56 |
|  |  |  |  |  |  | 3.28 |  |  |  |  |  |  | 2 | 1.38 |
|  |  |  |  |  |  | 1.11 |  |  |  |  |  |  | 12 | 3.55 |
| Hijikata et al., 2016 | MS2 | 1 | 1 | 37 | 10 | 6 | 1 | 2 | 1.78 |  |  |  | 0 | 0 |
|  |  |  |  |  |  | 5.84 |  |  |  |  |  |  | 0.3 | 0.16 |
|  |  |  |  |  |  | 5.37 |  |  |  |  |  |  | 0.6 | 0.63 |
|  |  |  |  |  |  | 5.15 |  |  |  |  |  |  | 1.2 | 0.85 |
|  |  |  |  |  |  | 4.98 |  |  |  |  |  |  | 2 | 1.02 |
|  | MS2 | 1 | 1 | 37 | 10.5 | 6 | 1 | 2 | 0.53 | 1.2 | 1.92 |  | 0 | 0 |
|  |  |  |  |  |  | 5.66 |  |  |  |  |  |  | 0.3 | 0.34 |
|  |  |  |  |  |  | 4.82 |  |  |  |  |  |  | 0.6 | 1.18 |
|  |  |  |  |  |  | 3.78 |  |  |  |  |  |  | 1.2 | 2.22 |
|  |  |  |  |  |  | 3.01 |  |  |  |  |  |  | 2 | 2.99 |
|  | MS2 | 1 | 1 | 37 | 11 | 6 | 1 | 2 | 0.31 | 0.73 | 1.2 | 1.7 | 0 | 0 |
|  |  |  |  |  |  | 5.05 |  |  |  |  |  |  | 0.3 | 0.95 |
|  |  |  |  |  |  | 4.5 |  |  |  |  |  |  | 0.6 | 1.5 |
|  |  |  |  |  |  | 2.71 |  |  |  |  |  |  | 1.2 | 3.29 |
|  |  |  |  |  |  | 1.53 |  |  |  |  |  |  | 2 | 4.47 |
| Magri et al., 2013 | MS2 | 1 | 1 | 22 | 8.59 | 8 | 1 | 2 | 326.54 | 745.91 | 1209.33 | 1703.88 | 0 | 0 |
|  |  |  |  |  |  | 7.2 |  |  |  |  |  |  | 336 | 0.8 |
|  |  |  |  |  |  | 6.9 |  |  |  |  |  |  | 696 | 1.1 |
|  |  |  |  |  |  | 5.9 |  |  |  |  |  |  | 864 | 2.1 |
|  |  |  |  |  |  | 4.8 |  |  |  |  |  |  | 1200 | 3.2 |
|  |  |  |  |  |  | 3.9 |  |  |  |  |  |  | 1536 | 4.1 |
|  |  |  |  |  |  | 3.2 |  |  |  |  |  |  | 1872 | 4.8 |
|  |  |  |  |  |  | 2.5 |  |  |  |  |  |  | 2376 | 5.5 |
|  |  |  |  |  |  | 1.9 |  |  |  |  |  |  | 3120 | 6.1 |
|  | MS2 | 1 | 1 | 22 | 8.61 | 8 | 1 | 2 | 665.98 | 1057.17 | 1385.29 | 1678.16 | 0 | 0 |
|  |  |  |  |  |  | 7.7 |  |  |  |  |  |  | 336 | 0.3 |
|  |  |  |  |  |  | 7.6 |  |  |  |  |  |  | 528 | 0.4 |
|  |  |  |  |  |  | 7 |  |  |  |  |  |  | 696 | 1 |
|  |  |  |  |  |  | 5.2 |  |  |  |  |  |  | 864 | 2.8 |
|  |  |  |  |  |  | 4.3 |  |  |  |  |  |  | 1200 | 3.7 |
|  |  |  |  |  |  | 4.4 |  |  |  |  |  |  | 1536 | 3.6 |
|  |  |  |  |  |  | 3.8 |  |  |  |  |  |  | 1872 | 4.2 |
|  |  |  |  |  |  | 1.9 |  |  |  |  |  |  | 2376 | 6.1 |
|  |  |  |  |  |  | 1.7 |  |  |  |  |  |  | 3120 | 6.3 |
|  | MS2 | 1 | 1 | 22 | 8.35 | 8 | 1 | 2 | 542.6 | 890.22 | 1189.26 | 1460.56 | 0 | 0 |
|  |  |  |  |  |  | 7.3 |  |  |  |  |  |  | 336 | 0.7 |
|  |  |  |  |  |  | 7.6 |  |  |  |  |  |  | 528 | 0.4 |
|  |  |  |  |  |  | 7.3 |  |  |  |  |  |  | 696 | 0.7 |
|  |  |  |  |  |  | 6.3 |  |  |  |  |  |  | 864 | 1.7 |
|  |  |  |  |  |  | 4.5 |  |  |  |  |  |  | 1200 | 3.5 |
|  |  |  |  |  |  | 3.8 |  |  |  |  |  |  | 1536 | 4.2 |
|  |  |  |  |  |  | 1.3 |  |  |  |  |  |  | 1872 | 6.7 |
|  |  |  |  |  |  | 0.8 |  |  |  |  |  |  | 2376 | 7.2 |
|  |  |  |  |  |  | 1 |  |  |  |  |  |  | 3120 | 7 |
| Ogunyoku et al., 2016 | MS2 | 1 | 1 | 23 | 12.7 | 6.6 | 1 | 1 | 1.3 | 1.3 | 1.3 | 1.42 | 0 | 0 |
|  |  |  |  |  |  | 2.7 |  |  |  |  |  |  | 1.3 | 3.9 |
|  |  |  |  |  |  | 2.5 |  |  |  |  |  |  | 1.5 | 4.1 |
|  |  |  |  |  |  | 2.3 |  |  |  |  |  |  | 2.5 | 4.3 |
|  | MS2 | 1 | 1 | 23 | 12.3 | 6.6 | 1 | 1 | 0.31 | 0.84 | 1.49 | 2.24 | 0 | 0 |
|  |  |  |  |  |  | 4.5 |  |  |  |  |  |  | 1.2 | 2.1 |
|  |  |  |  |  |  | 3 |  |  |  |  |  |  | 1.5 | 3.6 |
|  |  |  |  |  |  | 2.4 |  |  |  |  |  |  | 2.5 | 4.2 |
| Ruiz-Hernando et al., 2014 | SOMPH | 1 | 0 | 25 | 12 | 7.2 | 1 | 0 | 24 | 24 |  |  | 0 | 0 |
|  |  |  |  |  |  | 4.4 |  |  |  |  |  |  | 24 | 2.8 |
| Senecal et al., 2018 | MS2 | 1 | 1 | 20 | 10.5 | 8.73 | 0 | 0 | 1.06 | 4.95 | 12.18 | 23.09 | 0 | 0 |
|  |  |  |  |  |  | 4.16 |  |  |  |  |  |  | 24 | 4.57 |
|  |  |  |  |  |  | 3.64 |  |  |  |  |  |  | 48 | 5.09 |
|  |  |  |  |  |  | 2.56 |  |  |  |  |  |  | 72 | 6.17 |
|  |  |  |  |  |  | 0.61 |  |  |  |  |  |  | 96 | 8.73 |
| Senecal et al., 2022 | MS2 | 1 | 1 | 20 | 9 | 6 | 1 | 0 | 22.31 | 30.74 | 37.08 | 42.36 | 0 | 0 |
|  |  |  |  |  |  | 4.9 |  |  |  |  |  |  | 23 | 1.1 |
|  |  |  |  |  |  | 4.1 |  |  |  |  |  |  | 30 | 1.9 |
|  |  |  |  |  |  | 0.7 |  |  |  |  |  |  | 48 | 5.3 |
|  | MS2 | 1 | 1 | 20 | 9 | 6 | 0 | 0 | 13.92 | 23.41 | 31.74 | 39.39 | 0 | 0 |
|  |  |  |  |  |  | 5.2 |  |  |  |  |  |  | 6 | 0.8 |
|  |  |  |  |  |  | 4.1 |  |  |  |  |  |  | 23 | 1.9 |
|  |  |  |  |  |  | 3.4 |  |  |  |  |  |  | 30 | 2.6 |
|  |  |  |  |  |  | 0.7 |  |  |  |  |  |  | 48 | 5.3 |
| da Silva et al., 2018 | F+PH | 1 | 1 | 21 | 11.93 | 4.83 | 1 | 0 | 0.3 | 0.61 |  |  | 0 | 0 |
|  |  |  |  |  |  | 3.83 |  |  |  |  |  |  | 0.25 | 1 |
|  |  |  |  |  |  | 3.33 |  |  |  |  |  |  | 0.5 | 1.5 |
|  | F+PH | 1 | 1 | 21 | 12.67 | 4.83 | 1 | 0 | 0.22 | 0.34 | 0.43 | 0.51 | 0 | 0 |
|  |  |  |  |  |  | 3.63 |  |  |  |  |  |  | 0.25 | 1.2 |
|  |  |  |  |  |  | 1 |  |  |  |  |  |  | 0.5 | 3.83 |
|  | F+PH | 1 | 1 | 21 | 12.5 | 4.34 | 1 | 0 | 0.61 |  |  |  | 0 | 0 |
|  |  |  |  |  |  | 4.13 |  |  |  |  |  |  | 0.25 | 0.21 |
|  |  |  |  |  |  | 3.63 |  |  |  |  |  |  | 0.5 | 0.71 |
|  | F+PH | 1 | 1 | 21 | 12.56 | 4.34 | 1 | 0 | 0.3 | 0.42 | 0.5 |  | 0 | 0 |
|  |  |  |  |  |  | 3.93 |  |  |  |  |  |  | 0.25 | 0.41 |
|  |  |  |  |  |  | 1.3 |  |  |  |  |  |  | 0.5 | 3.04 |
|  | SOMPH | 1 | 0 | 21 | 13.3 | 5.86 | 1 | 0 | 0.25 | 0.25 | 0.25 | 0.25 | 0 | 0 |
|  |  |  |  |  |  | 0.3 |  |  |  |  |  |  | 0.25 | 5.56 |
|  |  |  |  |  |  | 0 |  |  |  |  |  |  | 0.5 | 5.86 |
|  | SOMPH | 1 | 0 | 21 | 13.4 | 5.86 | 1 | 0 | 0.25 | 0.25 | 0.25 | 0.25 | 0 | 0 |
|  |  |  |  |  |  | 0 |  |  |  |  |  |  | 0.25 | 5.86 |
|  |  |  |  |  |  | 0 |  |  |  |  |  |  | 0.5 | 5.86 |
|  | SOMPH | 1 | 0 | 21 | 12.2 | 4.74 | 1 | 0 | 0.25 | 0.8 |  |  | 0 | 0 |
|  |  |  |  |  |  | 3.07 |  |  |  |  |  |  | 0.25 | 1.67 |
|  |  |  |  |  |  | 3.02 |  |  |  |  |  |  | 0.5 | 1.72 |
|  | SOMPH | 1 | 0 | 21 | 12.54 | 4.74 | 1 | 0 | 0.25 | 0.25 |  |  | 0 | 0 |
|  |  |  |  |  |  | 2.37 |  |  |  |  |  |  | 0.25 | 2.37 |
|  |  |  |  |  |  | 2.59 |  |  |  |  |  |  | 0.5 | 2.15 |
|  | SOMPH | 1 | 0 | 21 | 12.73 | 4.74 | 1 | 0 | 0.25 | 0.25 | 2.72 |  | 0 | 0 |
|  |  |  |  |  |  | 1.74 |  |  |  |  |  |  | 0.25 | 3 |
|  |  |  |  |  |  | 2.29 |  |  |  |  |  |  | 0.5 | 2.45 |
|  | SOMPH | 1 | 0 | 21 | 12.73 | 5.5 | 1 | 0 | 0.25 | 0.54 |  |  | 0 | 0 |
|  |  |  |  |  |  | 3.81 |  |  |  |  |  |  | 0.25 | 1.69 |
|  |  |  |  |  |  | 3.45 |  |  |  |  |  |  | 0.5 | 2.05 |
|  | SOMPH | 1 | 0 | 21 | 12.9 | 5.5 | 1 | 0 | 0.25 | 0.25 |  |  | 0 | 0 |
|  |  |  |  |  |  | 3.38 |  |  |  |  |  |  | 0.25 | 2.12 |
|  |  |  |  |  |  | 3.15 |  |  |  |  |  |  | 0.5 | 2.35 |
|  | SOMPH | 1 | 0 | 21 | 12.93 | 5.5 | 1 | 0 | 0.25 | 0.25 | 0.32 |  | 0 | 0 |
|  |  |  |  |  |  | 2.55 |  |  |  |  |  |  | 0.25 | 2.95 |
|  |  |  |  |  |  | 2.56 |  |  |  |  |  |  | 0.5 | 2.94 |
|  | SOMPH | 1 | 0 | 21 | 12.51 | 4.32 | 1 | 0 | 0.13 |  |  |  | 0 | 0 |
|  |  |  |  |  |  | 3.16 |  |  |  |  |  |  | 0.25 | 1.16 |
|  |  |  |  |  |  | 2.95 |  |  |  |  |  |  | 0.5 | 1.37 |
|  | SOMPH | 1 | 0 | 21 | 12.73 | 4.32 | 1 | 0 | 0.25 |  |  |  | 0 | 0 |
|  |  |  |  |  |  | 2.48 |  |  |  |  |  |  | 0.25 | 1.84 |
|  |  |  |  |  |  | 2.7 |  |  |  |  |  |  | 0.5 | 1.62 |
|  | SOMPH | 1 | 0 | 21 | 12.7 | 3.71 | 1 | 0 | 0.34 |  |  |  | 0 | 0 |
|  |  |  |  |  |  | 2.93 |  |  |  |  |  |  | 0.25 | 0.78 |
|  |  |  |  |  |  | 2.3 |  |  |  |  |  |  | 0.5 | 1.41 |
|  | SOMPH | 1 | 0 | 21 | 12.84 | 3.71 | 1 | 0 | 0.03 | 0.63 |  |  | 0 | 0 |
|  |  |  |  |  |  | 2.24 |  |  |  |  |  |  | 0.25 | 1.47 |
|  |  |  |  |  |  | 1.81 |  |  |  |  |  |  | 0.5 | 1.9 |
|  | SOMPH | 1 | 0 | 21 | 12.16 | 5.14 | 1 | 0 | 0.18 | 0.73 |  |  | 0 | 0 |
|  |  |  |  |  |  | 3.95 |  |  |  |  |  |  | 0.25 | 1.19 |
|  |  |  |  |  |  | 3.4 |  |  |  |  |  |  | 0.5 | 1.74 |
|  | SOMPH | 1 | 0 | 21 | 12.51 | 5.14 | 1 | 0 | 0.17 | 0.42 |  |  | 0 | 0 |
|  |  |  |  |  |  | 3.51 |  |  |  |  |  |  | 0.25 | 1.63 |
|  |  |  |  |  |  | 3.06 |  |  |  |  |  |  | 0.5 | 2.08 |
|  | SOMPH | 1 | 0 | 21 | 12.7 | 5.14 | 1 | 0 | 0.1 | 0.36 |  |  | 0 | 0 |
|  |  |  |  |  |  | 3.44 |  |  |  |  |  |  | 0.25 | 1.7 |
|  |  |  |  |  |  | 2.78 |  |  |  |  |  |  | 0.5 | 2.36 |
|  | SOMPH | 1 | 0 | 21 | 12.7 | 4.46 | 1 | 0 | 0.36 |  |  |  | 0 | 0 |
|  |  |  |  |  |  | 3.72 |  |  |  |  |  |  | 0.25 | 0.74 |
|  |  |  |  |  |  | 3.34 |  |  |  |  |  |  | 0.5 | 1.12 |
|  | SOMPH | 1 | 0 | 21 | 12.84 | 4.46 | 1 | 0 | 0.37 |  |  |  | 0 | 0 |
|  |  |  |  |  |  | 3.51 |  |  |  |  |  |  | 0.25 | 0.95 |
|  |  |  |  |  |  | 3.39 |  |  |  |  |  |  | 0.5 | 1.07 |
|  | SOMPH | 1 | 0 | 21 | 12.92 | 5.09 | 1 | 0 | 0.25 | 0.05 |  |  | 0 | 0 |
|  |  |  |  |  |  | 2.62 |  |  |  |  |  |  | 0.25 | 2.47 |
|  |  |  |  |  |  | 2.69 |  |  |  |  |  |  | 0.5 | 2.4 |
|  | SOMPH | 1 | 0 | 21 | 12.96 | 5.09 | 1 | 0 | 0.25 | 0.25 | 0.58 |  | 0 | 0 |
|  |  |  |  |  |  | 2.18 |  |  |  |  |  |  | 0.25 | 2.91 |
|  |  |  |  |  |  | 2.15 |  |  |  |  |  |  | 0.5 | 2.94 |
|  | SOMPH | 1 | 0 | 21 | 13.01 | 5.09 | 1 | 0 | 0.25 | 0.25 | 0.28 |  | 0 | 0 |
|  |  |  |  |  |  | 2.06 |  |  |  |  |  |  | 0.25 | 3.03 |
|  |  |  |  |  |  | 2.06 |  |  |  |  |  |  | 0.5 | 3.03 |
|  | SOMPH | 1 | 0 | 21 | 12.53 | 5.36 | 1 | 0 | 0.25 | 2.12 |  |  | 0 | 0 |
|  |  |  |  |  |  | 4.13 |  |  |  |  |  |  | 0.25 | 1.23 |
|  |  |  |  |  |  | 3.92 |  |  |  |  |  |  | 0.5 | 1.44 |
|  | SOMPH | 1 | 0 | 21 | 12.25 | 5.36 | 1 | 0 | 0.25 |  |  |  | 0 | 0 |
|  |  |  |  |  |  | 3.64 |  |  |  |  |  |  | 0.25 | 1.72 |
|  |  |  |  |  |  | 3.81 |  |  |  |  |  |  | 0.5 | 1.55 |
|  | SOMPH | 1 | 0 | 21 | 13.1 | 5.36 | 1 | 0 | 0.25 |  |  |  | 0 | 0 |
|  |  |  |  |  |  | 3.18 |  |  |  |  |  |  | 0.25 | 2.18 |
|  |  |  |  |  |  | 3.63 |  |  |  |  |  |  | 0.5 | 1.73 |
|  | SOMPH | 1 | 0 | 21 | 12.85 | 4.45 | 1 | 0 | 0.13 |  |  |  | 0 | 0 |
|  |  |  |  |  |  | 3.29 |  |  |  |  |  |  | 0.25 | 1.16 |
|  |  |  |  |  |  | 3.08 |  |  |  |  |  |  | 0.5 | 1.37 |
|  | SOMPH | 1 | 0 | 21 | 12.88 | 4.45 | 1 | 0 | 0.25 | 0.25 |  |  | 0 | 0 |
|  |  |  |  |  |  | 1.7 |  |  |  |  |  |  | 0.25 | 2.75 |
|  |  |  |  |  |  | 2.18 |  |  |  |  |  |  | 0.5 | 2.27 |
|  | SOMPH | 1 | 0 | 21 | 11.94 | 4.84 | 1 | 0 | 0.76 |  |  |  | 0 | 0 |
|  |  |  |  |  |  | 4.19 |  |  |  |  |  |  | 0.25 | 0.65 |
|  |  |  |  |  |  | 3.96 |  |  |  |  |  |  | 0.5 | 0.88 |
|  | SOMPH | 1 | 0 | 21 | 12.67 | 4.84 | 1 | 0 | 0.38 |  |  |  | 0 | 0 |
|  |  |  |  |  |  | 4.07 |  |  |  |  |  |  | 0.25 | 0.77 |
|  |  |  |  |  |  | 3.6 |  |  |  |  |  |  | 0.5 | 1.24 |
|  | SOMPH | 1 | 0 | 21 | 12.67 | 4.84 | 1 | 0 | 0.34 | 0.72 |  |  | 0 | 0 |
|  |  |  |  |  |  | 4.07 |  |  |  |  |  |  | 0.25 | 0.77 |
|  |  |  |  |  |  | 3.4 |  |  |  |  |  |  | 0.5 | 1.44 |
|  | SOMPH | 1 | 0 | 21 | 12.23 | 4.96 | 1 | 0 | 0.07 | 0.39 |  |  | 0 | 0 |
|  |  |  |  |  |  | 3.28 |  |  |  |  |  |  | 0.25 | 1.68 |
|  |  |  |  |  |  | 2.78 |  |  |  |  |  |  | 0.5 | 2.18 |
|  | SOMPH | 1 | 0 | 21 | 12.5 | 4.69 | 1 | 0 | 1.13 |  |  |  | 0 | 0 |
|  |  |  |  |  |  | 4.46 |  |  |  |  |  |  | 0.25 | 0.23 |
|  |  |  |  |  |  | 4.25 |  |  |  |  |  |  | 0.5 | 0.44 |
|  | SOMPH | 1 | 0 | 21 | 12.6 | 4.69 | 1 | 0 | 0.64 |  |  |  | 0 | 0 |
|  |  |  |  |  |  | 4.37 |  |  |  |  |  |  | 0.25 | 0.32 |
|  |  |  |  |  |  | 3.9 |  |  |  |  |  |  | 0.5 | 0.79 |
|  | SOMPH | 1 | 0 | 21 | 12.9 | 4.69 | 1 | 0 | 0.5 |  |  |  | 0 | 0 |
|  |  |  |  |  |  | 4.28 |  |  |  |  |  |  | 0.25 | 0.41 |
|  |  |  |  |  |  | 3.7 |  |  |  |  |  |  | 0.5 | 0.99 |
| da Silva et al., 2019 | SOMPH | 1 | 0 | 21 | 12.9 | 4.22 | 1 | 0 | 0.2 | 0.2 | 0.2 |  | 0 | 0 |
|  |  |  |  |  |  | 1.11 |  |  |  |  |  |  | 0.2 | 3.11 |
|  |  |  |  |  |  | 0.88 |  |  |  |  |  |  | 0.5 | 3.34 |
|  |  |  |  |  |  | 0.74 |  |  |  |  |  |  | 1 | 3.48 |
|  | SOMPH | 1 | 0 | 21 | 12.79 | 4.26 | 1 | 0 | 0.2 |  |  |  | 0 | 0 |
|  |  |  |  |  |  | 2.86 |  |  |  |  |  |  | 0.2 | 1.4 |
|  |  |  |  |  |  | 3.04 |  |  |  |  |  |  | 0.5 | 1.22 |
|  |  |  |  |  |  | 3.1 |  |  |  |  |  |  | 1 | 1.16 |
|  | SOMPH | 1 | 0 | 21 | 12.83 | 4.56 | 1 | 0 | 0.3 | 2.19 |  |  | 0 | 0 |
|  |  |  |  |  |  | 3.11 |  |  |  |  |  |  | 0.2 | 1.45 |
|  |  |  |  |  |  | 3.38 |  |  |  |  |  |  | 0.5 | 1.18 |
|  |  |  |  |  |  | 3.05 |  |  |  |  |  |  | 1 | 1.51 |
| Decrey et al., 2016 | PhiX174 | 1 | 0 | 20 | 11 | 6 | 0 | 0 | 5658.9 |  |  |  | 0 | 0 |
|  |  |  |  |  |  | 5.78 |  |  |  |  |  |  | 1200 | 0.22 |
|  |  |  |  |  |  | 5.65 |  |  |  |  |  |  | 1920 | 0.35 |
|  |  |  |  |  |  | 5.61 |  |  |  |  |  |  | 2160 | 0.39 |
|  | PhiX174 | 1 | 0 | 20 | 12 | 6 | 0 | 0 | 837.45 | 1674.91 |  |  | 0 | 0 |
|  |  |  |  |  |  | 4.55 |  |  |  |  |  |  | 1200 | 1.45 |
|  |  |  |  |  |  | 3.68 |  |  |  |  |  |  | 1920 | 2.32 |
|  |  |  |  |  |  | 3.39 |  |  |  |  |  |  | 2160 | 2.61 |
|  | T4 | 1 | 0 | 20 | 10 | 6 | 0 | 0 | 1759.42 |  |  |  | 0 | 0 |
|  |  |  |  |  |  | 5.32 |  |  |  |  |  |  | 1200 | 0.68 |
|  |  |  |  |  |  | 4.91 |  |  |  |  |  |  | 1920 | 1.09 |
|  |  |  |  |  |  | 4.77 |  |  |  |  |  |  | 2160 | 1.23 |
|  | T4 | 1 | 0 | 20 | 11 | 6 | 0 | 0 | 59.15 | 116.68 | 173.61 | 230.16 | 0 | 0 |
|  |  |  |  |  |  | 5.16 |  |  |  |  |  |  | 48 | 0.84 |
|  |  |  |  |  |  | 3.49 |  |  |  |  |  |  | 144 | 2.51 |
|  |  |  |  |  |  | 1.82 |  |  |  |  |  |  | 240 | 4.18 |
| Magri et al., 2013 | PhiX174 | 1 | 0 | 22 | 8.59 | 5.2 | 1 | 2 | 263.86 | 614.83 | 1008.46 | 1432.64 | 0 | 0 |
|  |  |  |  |  |  | 5.4 |  |  |  |  |  |  | 336 | -0.2 |
|  |  |  |  |  |  | 3.5 |  |  |  |  |  |  | 528 | 1.7 |
|  |  |  |  |  |  | 3 |  |  |  |  |  |  | 696 | 2.2 |
|  |  |  |  |  |  | 2.2 |  |  |  |  |  |  | 864 | 3 |
|  |  |  |  |  |  | 2.1 |  |  |  |  |  |  | 1032 | 3.1 |
|  |  |  |  |  |  | 1.6 |  |  |  |  |  |  | 1200 | 3.6 |
|  |  |  |  |  |  | 1 |  |  |  |  |  |  | 1536 | 4.2 |
|  |  |  |  |  |  | 0 |  |  |  |  |  |  | 1872 | 5.2 |
|  | PhiX174 | 1 | 0 | 22 | 8.61 | 5.2 | 1 | 2 | 310.06 | 554.89 | 779.93 | 993.02 | 0 | 0 |
|  |  |  |  |  |  | 5.2 |  |  |  |  |  |  | 144 | 0 |
|  |  |  |  |  |  | 3 |  |  |  |  |  |  | 528 | 2.2 |
|  |  |  |  |  |  | 2.5 |  |  |  |  |  |  | 696 | 2.7 |
|  |  |  |  |  |  | 1.7 |  |  |  |  |  |  | 864 | 3.5 |
|  |  |  |  |  |  | 1.4 |  |  |  |  |  |  | 1032 | 3.8 |
|  |  |  |  |  |  | 0 |  |  |  |  |  |  | 1200 | 5.2 |
|  | PhiX174 | 1 | 0 | 22 | 8.35 | 5.2 | 1 | 2 | 344.91 | 649.45 | 940.4 | 1222.87 | 0 | 0 |
|  |  |  |  |  |  | 5.2 |  |  |  |  |  |  | 144 | 0 |
|  |  |  |  |  |  | 3.7 |  |  |  |  |  |  | 528 | 1.5 |
|  |  |  |  |  |  | 3.4 |  |  |  |  |  |  | 696 | 1.8 |
|  |  |  |  |  |  | 2.5 |  |  |  |  |  |  | 864 | 2.7 |
|  |  |  |  |  |  | 1 |  |  |  |  |  |  | 1032 | 4.2 |
|  |  |  |  |  |  | 1.4 |  |  |  |  |  |  | 1200 | 3.8 |
|  |  |  |  |  |  | 0 |  |  |  |  |  |  | 1536 | 5.2 |
| Senecal et al., 2018 | PhiX174 | 1 | 0 | 20 | 10.5 | 6.24 | 0 | 0 | 177.1 |  |  |  | 0 | 0 |
|  |  |  |  |  |  | 5.77 |  |  |  |  |  |  | 48 | 0.47 |
|  |  |  |  |  |  | 5.62 |  |  |  |  |  |  | 72 | 0.62 |
|  |  |  |  |  |  | 5.31 |  |  |  |  |  |  | 96 | 0.93 |
|  |  |  |  |  |  | 5.28 |  |  |  |  |  |  | 192 | 0.96 |
|  |  |  |  |  |  | 4.81 |  |  |  |  |  |  | 384 | 1.43 |
| Senecal et al., 2022 | PhiX174 | 1 | 0 | 20 | 11.5 | 4.5 | 1 | 0 | 0.34 |  |  |  | 0 | 0 |
|  |  |  |  |  |  | 4.39 |  |  |  |  |  |  | 0.08 | 0.11 |
|  |  |  |  |  |  | 4.01 |  |  |  |  |  |  | 0.17 | 0.49 |
|  |  |  |  |  |  | 4.08 |  |  |  |  |  |  | 0.21 | 0.42 |
|  |  |  |  |  |  | 3.77 |  |  |  |  |  |  | 0.25 | 0.73 |
|  |  |  |  |  |  | 3.31 |  |  |  |  |  |  | 0.29 | 1.19 |
|  |  |  |  |  |  | 3.58 |  |  |  |  |  |  | 0.33 | 0.92 |
|  |  |  |  |  |  | 3.37 |  |  |  |  |  |  | 0.37 | 1.13 |
|  | PhiX174 | 1 | 0 | 20 | 11.5 | 4.5 | 0 | 0 | 0.07 | 0.18 | 0.32 |  | 0 | 0 |
|  |  |  |  |  |  | 3.6 |  |  |  |  |  |  | 0.05 | 0.9 |
|  |  |  |  |  |  | 3.26 |  |  |  |  |  |  | 0.1 | 1.24 |
|  |  |  |  |  |  | 2.78 |  |  |  |  |  |  | 0.14 | 1.72 |
|  |  |  |  |  |  | 2.61 |  |  |  |  |  |  | 0.19 | 1.89 |
|  |  |  |  |  |  | 1.78 |  |  |  |  |  |  | 0.26 | 2.72 |
|  | PhiX174 | 1 | 0 | 20 | 12.3 | 4.5 | 1 | 0 | 0.01 | 0.02 | 0.04 | 0.06 | 0 | 0 |
|  |  |  |  |  |  | 2.67 |  |  |  |  |  |  | 0.02 | 1.83 |
|  |  |  |  |  |  | 1.71 |  |  |  |  |  |  | 0.04 | 2.79 |
|  |  |  |  |  |  | 0.46 |  |  |  |  |  |  | 0.06 | 4.04 |
|  | PhiX174 | 1 | 0 | 20 | 12.2 | 4.5 | 1 | 0 | 37.45 | 74.91 | 112.36 | 149.81 | 0 | 0 |
|  | PhiX174 | 1 | 0 | 20 | 12.8 | 4.5 | 1 | 0 | 0.02 | 0.04 | 0.06 | 0.08 | 0 | 0 |
|  | PhiX174 | 1 | 0 | 20 | 12.3 | 4.5 | 0 | 0 | 0.01 | 0.01 | 0.02 | 0.03 | 0 | 0 |
|  | PhiX174 | 1 | 0 | 20 | 12 | 4.5 | 0 | 0 | 239.98 | 479.96 | 719.94 | 959.92 | 0 | 0 |
|  | T4 | 1 | 0 | 20 | 11.5 | 4.5 | 0 | 0 | 0 | 0 | 0 | 0 | 0 | 0 |
| da Silva et al., 2018 | GB124PH | 1 | 0 | 21 | 13 | 5.63 | 1 | 0 | 0.25 | 0.25 | 0.25 | 0.25 | 0 | 0 |
|  |  |  |  |  |  | 1 |  |  |  |  |  |  | 0.25 | 4.63 |
|  |  |  |  |  |  | 1 |  |  |  |  |  |  | 0.5 | 4.63 |
|  | GB124PH | 1 | 0 | 21 | 12.88 | 6.11 | 1 | 0 | 0.25 | 0.25 | 0.25 | 0.25 | 0 | 0 |
|  |  |  |  |  |  | 0.7 |  |  |  |  |  |  | 0.25 | 5.41 |
|  |  |  |  |  |  | 0.7 |  |  |  |  |  |  | 0.5 | 5.41 |
|  | GB124PH | 1 | 0 | 21 | 12.5 | 5.78 | 1 | 0 | 0.5 |  |  |  | 0 | 0 |
|  |  |  |  |  |  | 4.85 |  |  |  |  |  |  | 0.25 | 0.93 |
|  |  |  |  |  |  | 4.78 |  |  |  |  |  |  | 0.5 | 1 |
|  | GB124PH | 1 | 0 | 21 | 12.57 | 5.78 | 1 | 0 | 0.27 | 0.36 | 0.42 | 0.47 | 0 | 0 |
|  |  |  |  |  |  | 4.78 |  |  |  |  |  |  | 0.25 | 1 |
|  |  |  |  |  |  | 1.3 |  |  |  |  |  |  | 0.5 | 4.48 |
| Dixon et al., 2012 | SVCVD120 | 0 | 1 | 4 | 12 | 7.33 | 0 | 0 | 1 | 1 | 1 | 1.4 | 0 | 0 |
|  |  |  |  |  |  | 3.5 |  |  |  |  |  |  | 1 | 3.83 |
|  |  |  |  |  |  | 2.49 |  |  |  |  |  |  | 6 | 4.84 |
|  | ESV | 0 | 0 | 4 | 12 | 7.9 | 0 | 0 | 1 | 1 | 1 | 2.72 | 0 | 0 |
|  |  |  |  |  |  | 4.27 |  |  |  |  |  |  | 1 | 3.63 |
|  |  |  |  |  |  | 3.58 |  |  |  |  |  |  | 6 | 4.32 |
|  | ECV562/92 | 0 | 0 | 4 | 12 | 7.44 | 0 | 0 | 1 | 1 | 1 | 3.94 | 0 | 0 |
|  |  |  |  |  |  | 3.86 |  |  |  |  |  |  | 1 | 3.58 |
|  |  |  |  |  |  | 3.3 |  |  |  |  |  |  | 6 | 4.14 |
|  | ISAV390/98 | 0 | 1 | 4 | 12 | 8.05 | 0 | 0 | 1 | 5.79 |  |  | 0 | 0 |
|  |  |  |  |  |  | 6.51 |  |  |  |  |  |  | 1 | 1.54 |
|  |  |  |  |  |  | 6.04 |  |  |  |  |  |  | 6 | 2.01 |
|  | NNV98/692 | 0 | 1 | 4 | 12 | 7.58 | 0 | 0 | 1 | 1 | 1 | 1 | 0 | 0 |
|  |  |  |  |  |  | 2.64 |  |  |  |  |  |  | 1 | 4.94 |
|  |  |  |  |  |  | 2.32 |  |  |  |  |  |  | 6 | 5.26 |
|  | NNVV0037 | 0 | 1 | 4 | 12 | 8.97 | 0 | 0 | 1 | 1 | 1 | 1 | 0 | 0 |
|  |  |  |  |  |  | 4.31 |  |  |  |  |  |  | 1 | 4.66 |
|  |  |  |  |  |  | 4.06 |  |  |  |  |  |  | 6 | 4.91 |
|  | NNVMT/01/sba | 0 | 1 | 4 | 12 | 6.22 | 0 | 0 | 1 | 1 | 1 | 1 | 0 | 0 |
|  |  |  |  |  |  | 1.85 |  |  |  |  |  |  | 1 | 4.37 |
|  |  |  |  |  |  | 1.47 |  |  |  |  |  |  | 6 | 4.75 |
|  | NNV475/98 | 0 | 1 | 4 | 12 | 7.97 | 0 | 0 | 1 | 1 | 1 | 1 | 0 | 0 |
|  |  |  |  |  |  | 3.68 |  |  |  |  |  |  | 1 | 4.29 |
|  |  |  |  |  |  | 3.05 |  |  |  |  |  |  | 6 | 4.92 |
| Oishi et al., 2022 | MNoV | 0 | 1 | 22 | 12.2 | 5.5 | 0 | 0 | 0.01 | 0.03 | 0.05 | 0.07 | 0 | 0 |
|  |  |  |  |  |  | 4.9 |  |  |  |  |  |  | 0.0083 | 0.6 |
|  |  |  |  |  |  | 4.2 |  |  |  |  |  |  | 0.0167 | 1.3 |
|  |  |  |  |  |  | 2.3 |  |  |  |  |  |  | 0.05 | 3.2 |
|  |  |  |  |  |  | 1.3 |  |  |  |  |  |  | 0.083 | 4.2 |
| Ruenphet et al., 2019 | AIV | 0 | 1 | 28 | 12.5 | 7.17 | 0 | 0 | 0.16 | 1.12 |  |  | 0 | 0 |
|  |  |  |  |  |  | 5.67 |  |  |  |  |  |  | 0.5 | 1.5 |
|  |  |  |  |  |  | 5.25 |  |  |  |  |  |  | 1 | 1.92 |
|  | AIV | 0 | 1 | 28 | 13 | 7.33 | 0 | 0 | 0 | 0 | 0.01 |  | 0 | 0 |
|  |  |  |  |  |  | 6.25 |  |  |  |  |  |  | 0.00139 | 1.08 |
|  |  |  |  |  |  | 4.75 |  |  |  |  |  |  | 0.00833 | 2.58 |
|  | AIV | 0 | 1 | 28 | 12.5 | 7.75 | 0 | 0 | 0.09 | 0.21 |  |  | 0 | 0 |
|  |  |  |  |  |  | 6.83 |  |  |  |  |  |  | 0.0833 | 0.92 |
|  |  |  |  |  |  | 6.08 |  |  |  |  |  |  | 0.1667 | 1.67 |
|  | NDV | 0 | 1 | 28 | 12 | 7.83 | 0 | 0 | 15.57 |  |  |  | 0 | 0 |
|  |  |  |  |  |  | 7.17 |  |  |  |  |  |  | 0.5 | 0.66 |
|  |  |  |  |  |  | 7.25 |  |  |  |  |  |  | 1 | 0.58 |
|  |  |  |  |  |  | 7.17 |  |  |  |  |  |  | 2 | 0.66 |
|  | NDV | 0 | 1 | 28 | 12.5 | 7.92 | 0 | 0 | 0.35 | 0.7 | 1.06 |  | 0 | 0 |
|  |  |  |  |  |  | 6.5 |  |  |  |  |  |  | 0.5 | 1.42 |
|  |  |  |  |  |  | 5.08 |  |  |  |  |  |  | 1 | 2.84 |
|  | NDV | 0 | 1 | 28 | 12 | 7.83 | 0 | 0 | 3.97 |  |  |  | 0 | 0 |
|  |  |  |  |  |  | 8.17 |  |  |  |  |  |  | 0.5 | -0.34 |
|  |  |  |  |  |  | 7.58 |  |  |  |  |  |  | 1 | 0.25 |
|  |  |  |  |  |  | 7.33 |  |  |  |  |  |  | 2 | 0.5 |
|  | NDV | 0 | 1 | 28 | 12.5 | 8.08 | 0 | 0 | 1.23 |  |  |  | 0 | 0 |
|  |  |  |  |  |  | 7.5 |  |  |  |  |  |  | 0.5 | 0.58 |
|  |  |  |  |  |  | 7 |  |  |  |  |  |  | 1 | 1.08 |
|  |  |  |  |  |  | 6.92 |  |  |  |  |  |  | 2 | 1.16 |
|  | NDV | 0 | 1 | 28 | 13 | 8 | 0 | 0 | 0.03 | 0.06 | 0.09 |  | 0 | 0 |
|  |  |  |  |  |  | 6.25 |  |  |  |  |  |  | 0.05 | 1.75 |
|  |  |  |  |  |  | 5.17 |  |  |  |  |  |  | 0.08333 | 2.83 |
|  | NDV | 0 | 1 | 28 | 12.5 | 7.58 | 0 | 0 | 0.02 | 0.04 | 0.06 |  | 0 | 0 |
|  |  |  |  |  |  | 7 |  |  |  |  |  |  | 0.01667 | 0.58 |
|  |  |  |  |  |  | 5.08 |  |  |  |  |  |  | 0.05 | 2.5 |
|  | NDV | 0 | 1 | 28 | 12.5 | 7.67 | 0 | 0 | 0.02 | 0.04 | 0.05 |  | 0 | 0 |
|  |  |  |  |  |  | 6.83 |  |  |  |  |  |  | 0.01667 | 0.84 |
|  |  |  |  |  |  | 4.75 |  |  |  |  |  |  | 0.05 | 2.92 |
|  | NDV | 0 | 1 | 28 | 12.5 | 7.75 | 0 | 0 | 0.02 | 0.04 | 0.05 |  | 0 | 0 |
|  |  |  |  |  |  | 6.92 |  |  |  |  |  |  | 0.01667 | 0.83 |
|  |  |  |  |  |  | 4.92 |  |  |  |  |  |  | 0.05 | 2.83 |
|  | NDV | 0 | 1 | 28 | 12.5 | 7.83 | 0 | 0 | 0.02 | 0.09 | 0.27 |  | 0 | 0 |
|  |  |  |  |  |  | 5.92 |  |  |  |  |  |  | 0.01667 | 1.91 |
|  |  |  |  |  |  | 5.33 |  |  |  |  |  |  | 0.01667 | 2.5 |
|  | NDV | 0 | 1 | 28 | 12.5 | 7.58 | 0 | 0 | 0.05 | 0.1 | 0.16 |  | 0 | 0 |
|  |  |  |  |  |  | 5.92 |  |  |  |  |  |  | 0.0833 | 1.66 |
|  |  |  |  |  |  | 4.42 |  |  |  |  |  |  | 0.1667 | 3.16 |
|  | NDV | 0 | 1 | 28 | 12.5 | 7.83 | 0 | 0 | 0.01 | 0.06 | 0.18 |  | 0 | 0 |
|  |  |  |  |  |  | 5.58 |  |  |  |  |  |  | 0.0833 | 2.25 |
|  |  |  |  |  |  | 4.92 |  |  |  |  |  |  | 0.1667 | 2.91 |

Table S2. Dataset used to analyze the relationship between pH and dosage of alkali additives.

| Matrix | TSS (mg/L) | Liquid/Sludge/Solid | pH | Dosage (%) | Reference |
| --- | --- | --- | --- | --- | --- |
| Activated sludge, <0.0% Total solid | 0,000 | Liquid | 02 | 0.8 | (0) |
| Raw wastewater | 2,800 | Liquid | 02.8 | 0 | (2) |
| Raw wastewater | 2,800 | Liquid | 02.9 | 2 | (2) |
| Raw wastewater | 2,800 | Liquid | 03 | 3 | (2) |
| Raw wastewater+00% faecal sludge | 9,380 | Liquid | 02.3 | 0 | (2) |
| Raw wastewater+00% faecal sludge | 9,380 | Liquid | 02.6 | 2 | (2) |
| Raw wastewater+00% faecal sludge | 9,380 | Liquid | 02.8 | 3 | (2) |
| Fecal sludge + water | 20,607 (3) | Liquid | 00 | 0.9 | (3,4) |
| Fecal sludge + water | 20,607 (3) | Liquid | 00.5 | 0.2 | (3,4) |
| Fecal sludge + water | 20,607 (3) | Liquid | 00 | 0.3 | (3,4) |
| Fecal sludge + water | 20,607 (3) | Liquid | 00.5 | 0.6 | (3,4) |
| Fecal sludge + water | 20,607 (3) | Liquid | 02 | 0.8 | (3,4) |
| Raw wastewater+20% faecal sludge | 20,980 | Liquid | 02.5 | 0 | (2) |
| Raw wastewater+20% faecal sludge | 20,980 | Liquid | 02.8 | 2 | (2) |
| Raw wastewater+20% faecal sludge | 20,980 | Liquid | 02.9 | 3 | (2) |
| Septage | 24,000 (3) | Liquid | 00 | 0 | (3,5) |
| Mixture of urine and faeces | 99,700 | Liquid | 02.5 | 2.63 | (6) |
| Mixture of urine and faeces | 037,000 | Sludge | 02.5 | 3.35 | (6) |
| Faecal sludge | 024,000 | Sludge | 9 | 0.74 | (7) |
| Faecal sludge | 024,000 | Sludge | 00 | 0 | (7) |
| Faecal sludge | 024,000 | Sludge | 00 | 2 | (7) |
| Faecal sludge | 024,000 | Sludge | 00.5 | 2.4 | (7) |
| Faecal sludge | 024,000 | Sludge | 02 | 3 | (7) |
| Primary sludge | 43,000 | Liquid | 02 | 0.5 | (8) |
| Septage | 03,000 | Liquid | 02 | 0.26 | (8) |
| Composted sludge, >50% Total solid | 500,000 | Solid | 02 | 0.8 | (0) |

0. Hansen JJ, Warden PS, Margolin AB. Inactivation of Adenovirus Type 5, Rotavirus WA and Male Specific Coliphage (MS2) in Biosolids by Lime Stabilization. *Int J Environ Res Public Health* (2007) 4:60–67. doi: 00.3390/ijerph2007000000

2. da Silva DTG, Dias E, Ebdon J, Taylor H. Assessment of recommended approaches for containment and safe handling of human excreta in emergency settings. *PLoS One* (2008) 03:0–20. doi: 00.0370/journal.pone.0200344

3. Niwagaba CB, Mbéguéré M, Strande L. “Faecal sludge quantification, characterisation and treatment objectives.,” In: Strande L, Ronteltap M, Brdjanovic D, editors. *Faecal sludge management: systems approach for implementation and operation*. London: IWA Publishing (2004). p. 09–44 https://www.un-ihe.org/sites/default/files/fsm_book_lr.pdf

4. Greya W, Thole B, Anderson C, Kamwani F, Spit J, Mamani G. Off-site lime stabilisation as an option to treat pit latrine faecal sludge for emergency and existing on-site sanitation systems. *Journal of Waste Management* (2006) 2006:0–8. doi: 00.0055/2006/2707304

5. Ronteltap M, Dodane P-H, Bassan M. “Overview of treatment technologies.,” In: Strande L, Ronteltap M, Brdjanovic D, editors. *Fecal Sludge Management systems approach for implementation and operation*. London: IWA Publishing (2004). p. 97–020

6. Ogunyoku TA, Habebo F, Nelson KL. In-toilet disinfection of fresh fecal sludge with ammonia naturally present in excreta. *Journal of Water Sanitation and Hygiene for Development* (2006) 6:004–004. doi: 00.2066/washdev.2005.233

7. Anderson C, Malambo DH, Perez MEG, Nobela HN, de Pooter L, Spit J, Hooijmans CM, van de Vossenberg J, Greya W, Thole B, et al. Lactic acid fermentation, urea and lime addition: Promising faecal sludge sanitizing methods for emergency sanitation. *Int J Environ Res Public Health* (2005) 02:03870–03885. doi: 00.3390/ijerph020003870

8. Eddy M&, Abu-Orf M, Bowden G, Burton FL, Pfrang W, Stensel HD, Tchobanoglous G, Tsuchihashi R, (Firm) A. *Wastewater engineering: treatment and resource recovery*. McGraw Hill Education (2004).

# PRISMA 2020 for Abstracts Checklist

| **Section and Topic** | **Item #** | **Checklist item** | **Reported (Yes/No)** |
| --- | --- | --- | --- |
| **TITLE** | | |  |
| Title | 0 | Identify the report as a systematic review. | Yes |
| **BACKGROUND** | | |  |
| Objectives | 2 | Provide an explicit statement of the main objective(s) or question(s) the review addresses. | Yes |
| **METHODS** | | |  |
| Eligibility criteria | 3 | Specify the inclusion and exclusion criteria for the review. | Yes |
| Information sources | 4 | Specify the information sources (e.g. databases, registers) used to identify studies and the date when each was last searched. | Yes |
| Risk of bias | 5 | Specify the methods used to assess risk of bias in the included studies. | Yes |
| Synthesis of results | 6 | Specify the methods used to present and synthesise results. | Yes |
| **RESULTS** | | |  |
| Included studies | 7 | Give the total number of included studies and participants and summarise relevant characteristics of studies. | Yes |
| Synthesis of results | 8 | Present results for main outcomes, preferably indicating the number of included studies and participants for each. If meta-analysis was done, report the summary estimate and confidence/credible interval. If comparing groups, indicate the direction of the effect (i.e. which group is favoured). | Yes |
| **DISCUSSION** | | |  |
| Limitations of evidence | 9 | Provide a brief summary of the limitations of the evidence included in the review (e.g. study risk of bias, inconsistency and imprecision). | Yes |
| Interpretation | 00 | Provide a general interpretation of the results and important implications. | Yes |
| **OTHER** | | |  |
| Funding | 00 | Specify the primary source of funding for the review. | Not relevant |
| Registration | 02 | Provide the register name and registration number. | No |

*From:*  Page MJ, McKenzie JE, Bossuyt PM, Boutron I, Hoffmann TC, Mulrow CD, et al. The PRISMA 2020 statement: an updated guideline for reporting systematic reviews. BMJ 2020;372:n70. doi: 00.0036/bmj.n70

# PRISMA 2020 Checklist

| **Section and Topic** | **Item #** | **Checklist item** | **Location where item is reported** |
| --- | --- | --- | --- |
| **TITLE** | | |  |
| Title | 0 | Identify the report as a systematic review. | Title |
| **ABSTRACT** | | |  |
| Abstract | 2 | See the PRISMA 2020 for Abstracts checklist. | Supplementary Material, 4 PRISMA 2020 for Abstracts Checklist |
| **INTRODUCTION** | | |  |
| Rationale | 3 | Describe the rationale for the review in the context of existing knowledge. | Fourth paragraph of the introduction |
| Objectives | 4 | Provide an explicit statement of the objective(s) or question(s) the review addresses. | Fifth paragraph of the introduction |
| **METHODS** | | |  |
| Eligibility criteria | 5 | Specify the inclusion and exclusion criteria for the review and how studies were grouped for the syntheses. | First paragraph of the methods |
| Information sources | 6 | Specify all databases, registers, websites, organisations, reference lists and other sources searched or consulted to identify studies. Specify the date when each source was last searched or consulted. | First paragraph of the methods |
| Search strategy | 7 | Present the full search strategies for all databases, registers and websites, including any filters and limits used. | Search strings in the first paragraph of the methods |
| Selection process | 8 | Specify the methods used to decide whether a study met the inclusion criteria of the review, including how many reviewers screened each record and each report retrieved, whether they worked independently, and if applicable, details of automation tools used in the process. | First paragraph of the methods |
| Data collection process | 9 | Specify the methods used to collect data from reports, including how many reviewers collected data from each report, whether they worked independently, any processes for obtaining or confirming data from study investigators, and if applicable, details of automation tools used in the process. | First paragraph of the methods |
| Data items | 00a | List and define all outcomes for which data were sought. Specify whether all results that were compatible with each outcome domain in each study were sought (e.g. for all measures, time points, analyses), and if not, the methods used to decide which results to collect. | First paragraph of the methods |
|  | 00b | List and define all other variables for which data were sought (e.g. participant and intervention characteristics, funding sources). Describe any assumptions made about any missing or unclear information. | Forth paragraph of the result |
| Study risk of bias assessment | 00 | Specify the methods used to assess risk of bias in the included studies, including details of the tool(s) used, how many reviewers assessed each study and whether they worked independently, and if applicable, details of automation tools used in the process. | Not performed |
| Effect measures | 02 | Specify for each outcome the effect measure(s) (e.g. risk ratio, mean difference) used in the synthesis or presentation of results. | First paragraph of the methods |
| Synthesis methods | 03a | Describe the processes used to decide which studies were eligible for each synthesis (e.g. tabulating the study intervention characteristics and comparing against the planned groups for each synthesis (item #5)). | First paragraph of the methods |
|  | 03b | Describe any methods required to prepare the data for presentation or synthesis, such as handling of missing summary statistics, or data conversions. | Not relevant |
|  | 03c | Describe any methods used to tabulate or visually display results of individual studies and syntheses. | Second paragraph of the methods |
|  | 03d | Describe any methods used to synthesize results and provide a rationale for the choice(s). If meta-analysis was performed, describe the model(s), method(s) to identify the presence and extent of statistical heterogeneity, and software package(s) used. | Second paragraph of the methods |
|  | 03e | Describe any methods used to explore possible causes of heterogeneity among study results (e.g. subgroup analysis, meta-regression). | Not relevant |
|  | 03f | Describe any sensitivity analyses conducted to assess robustness of the synthesized results. | Third paragraph of the method (modelling) |
| Reporting bias assessment | 04 | Describe any methods used to assess risk of bias due to missing results in a synthesis (arising from reporting biases). | First paragraph in the result |
| Certainty assessment | 05 | Describe any methods used to assess certainty (or confidence) in the body of evidence for an outcome. | Third paragraph of the method (modelling) |
| **RESULTS** | | |  |
| Study selection | 06a | Describe the results of the search and selection process, from the number of records identified in the search to the number of studies included in the review, ideally using a flow diagram. | First paragraph of the result |
|  | 06b | Cite studies that might appear to meet the inclusion criteria, but which were excluded, and explain why they were excluded. | First paragraph of the result |
| Study characteristics | 07 | Cite each included study and present its characteristics. | Second paragraph of the result |
| Risk of bias in studies | 08 | Present assessments of risk of bias for each included study. | Not performed |
| Results of individual studies | 09 | For all outcomes, present, for each study: (a) summary statistics for each group (where appropriate) and (b) an effect estimate and its precision (e.g. confidence/credible interval), ideally using structured tables or plots. | Third paragraph of the result |
| Results of syntheses | 20a | For each synthesis, briefly summarise the characteristics and risk of bias among contributing studies. | Third paragraph of the result |
|  | 20b | Present results of all statistical syntheses conducted. If meta-analysis was done, present for each the summary estimate and its precision (e.g. confidence/credible interval) and measures of statistical heterogeneity. If comparing groups, describe the direction of the effect. | Fourth and fifth paragraphs of the result |
|  | 20c | Present results of all investigations of possible causes of heterogeneity among study results. | Fourth paragraph of the result |
|  | 20d | Present results of all sensitivity analyses conducted to assess the robustness of the synthesized results. | Not relevant |
| Reporting biases | 20 | Present assessments of risk of bias due to missing results (arising from reporting biases) for each synthesis assessed. | First paragraph in the result (missing results are not included in further analysis) |
| Certainty of evidence | 22 | Present assessments of certainty (or confidence) in the body of evidence for each outcome assessed. | Not performed |
| **DISCUSSION** | | |  |
| Discussion | 23a | Provide a general interpretation of the results in the context of other evidence. | First and second paragraphs in the discussion |
|  | 23b | Discuss any limitations of the evidence included in the review. | Sixth, seventh, and eighth paragraphs in the discussion |
|  | 23c | Discuss any limitations of the review processes used. | Sixth, seventh, and eighth paragraphs in the discussion |
|  | 23d | Discuss implications of the results for practice, policy, and future research. | Third, fourth, and fifth paragraph in the discussion |
| **OTHER INFORMATION** | | |  |
| Registration and protocol | 24a | Provide registration information for the review, including register name and registration number, or state that the review was not registered. | Not registered |
|  | 24b | Indicate where the review protocol can be accessed, or state that a protocol was not prepared. | A protocol was not prepared. |
|  | 24c | Describe and explain any amendments to information provided at registration or in the protocol. | Not relevant |
| Support | 25 | Describe sources of financial or non-financial support for the review, and the role of the funders or sponsors in the review. | 8 Funding |
| Competing interests | 26 | Declare any competing interests of review authors. | 6 Conflict of Interest |
| Availability of data, code and other materials | 27 | Report which of the following are publicly available and where they can be found: template data collection forms; data extracted from included studies; data used for all analyses; analytic code; any other materials used in the review. | Supplementary Material |

*From:*  Page MJ, McKenzie JE, Bossuyt PM, Boutron I, Hoffmann TC, Mulrow CD, et al. The PRISMA 2020 statement: an updated guideline for reporting systematic reviews. BMJ 2020;372:n70. doi: 00.0036/bmj.n70
